# Epigenetic dysregulation in depression: molecular mechanisms, clinical biomarkers, and therapeutic opportunities

**DOI:** 10.1186/s12967-026-08772-0

**Published:** 2026-08-08

**Authors:** Jiayu Li, Xuchu Guan, Ruigang Zhang, Huahua Zhang

**Affiliations:** 1 Department of Physiology, School of Basic Medical Sciences, Guangdong Medical University, Dongguan, Guangdong 523808 China; 2 Department of Biology, School of Basic Medical Sciences, Guangdong Medical University, Zhanjiang, Guangdong 524023 China; 3 Department of Physiology, School of Basic Medical Sciences, Guangdong Medical University, Zhanjiang, Guangdong 524023 China; 4 Department of Biology, School of Basic Medical Sciences, Guangdong Medical University, Dongguan, Guangdong 523808 China

**Keywords:** Depression, Epigenetics, DNA methylation, Histone modification, Non-coding RNA, RNA modification, Chromatin remodeling, Epigenetic clocks

## Abstract

Major depressive disorder (MDD) is a widespread, recurrent, and severely disabling psychiatric disorder that imposes a heavy global health burden. Although genetic factors contribute to disease risk, growing evidence highlights gene-environment interaction as the core driver of MDD pathogenesis. Epigenetic regulation acts as a precisely molecular interface that translates environmental stressors into stable changes in gene expression and long-term behavioral phenotypes. In this review, we provide a comprehensive and up-to-date overview of epigenetic dysregulation in MDD, covering five major regulatory layers: DNA methylation, histone post-translational modifications, non-coding RNA networks, RNA chemical modifications, and ATP-dependent chromatin remodeling. We emphasize the spatiotemporal specificity, brain regional selectivity, and cell-type. dependency of these epigenetic events, and their roles in disrupting neuroplasticity, hypothalamic–pituitary–adrenal (HPA) axis function, neurotransmitter homeostasis, and neuroinflammation. We further evaluate the translational value of peripheral epigenetic markers for early diagnosis, severity monitoring, and prediction of antidepressant treatment responses. We also discuss emerging epigenetic-targeted therapeutic strategies, including small-molecule inhibitors, RNA-based modulators, and brain-targeted delivery systems. Finally, we address key obstacles to clinical translation, such as tissue heterogeneity, unclear causality, limited reproducibility, and lack of standardized protocols. We propose future directions centered on single-cell multi-omics, longitudinal clinical validation, and sex-and ethnicity-stratified research. This review aims to establish an integrated framework for understanding MDD epigenetics and accelerating the development of precision diagnostic and therapeutic approaches.

## Introduction

Major depressive disorder (MDD) is a leading global cause of disability, affecting more than 332 million people and creating a major public health challenge [1]. Core clinical features include persistent low mood, anhedonia, cognitive dysfunction, and increased suicide risk [2]. Epidemiological studies reveal clear sex and age differences: the lifetime prevalence in women reaches 4.1%, roughly 1.5 times that in men (2.7%). People aged 50 years and above also face a 1.5-fold higher risk than those aged 18–29 years. Notably, nearly 50% of patients relapse within one year after remission, confirming the chronic and recurrent nature of MDD and the urgent demand to disentangle its multifactorial pathogenic network [3].

The term “epigenetics” was first introduced by Waddington in 1942 to describe developmental processes that convert genotype into phenotype. With advances in molecular biology, epigenetics is now defined as heritable changes in gene expression that do not alter the underlying DNA sequence. Its core regulatory mechanisms include DNA methylation, histone modifications, and non-coding RNA regulation. These pathways are highly dynamic and sensitive to environmental stimuli, and they serve essential functions in neurodevelopment and stress adaptation [4].

Traditional genetic research suggests that the heritability of MDD ranges from 31% to 42% [5]. However, the concordance rate in monozygotic twins is only about 50%.meaning genetic factors alone cannot fully account for MDD pathogenesis [6]. As the gene-environment interaction model has become widely accepted, researchers recognize that interactions between environmental stressors (such as childhood trauma, chronic stress, and prenatal adverse events) and genetic background contribute critically to disease vulnerability [7]. Individuals differ greatly in stress resilience: some remain mentally stable under long-term stress, while others develop obvious depressive symptoms. The molecular basis of this heterogeneity remains a key scientific question [8]. Therefore, MDD pathogenesis is multifactorial, involving complex interplay among genetic vulnerability, environmental exposures, neurobiological alterations, and psychosocial factors. Within this multidimensional framework, epigenetics represents a critical molecular interface that integrates environmental signals with genomic outputs, translating external stimuli into sustained transcriptional and behavioral changes.

**Fig. 1:**
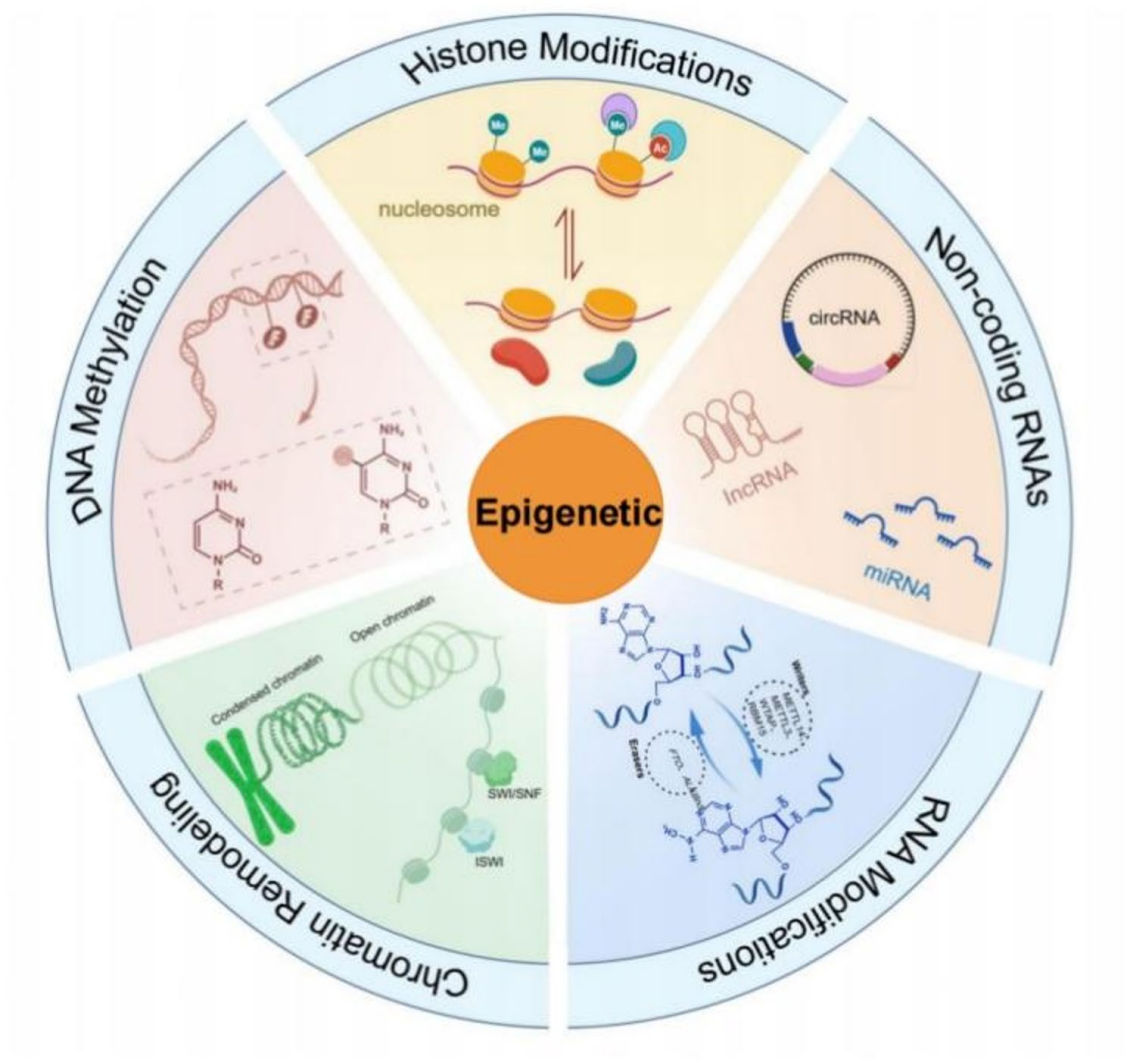
Schematic diagram of five core epigenetic regulatory mechanisms implicated in major depressive disorder (MDD).This illustration depicts the principal epigenetic layers: **(a)** DNA methylation, which governs gene transcription by mediating cytosine methylation; **(b)** Histone modifications, which reshape chromatin compaction through post-translational marks including acetylation and methylation; **(c)** Non-coding RNAs, encompassing miRNAs, circRNAs, and lncRNAs, which exert post-transcriptional regulatory functions; **(d)** RNA modifications, predominantly N⁶-methyladenosine (m⁶A), which orchestrate RNA turnover, stability, and biological functions; **(e)** Chromatin remodeling, which tunes chromatin accessibility via multi-subunit remodeling complexes. Collectively, these epigenetic events bridge environmental stressors to transcriptional reprogramming and are tightly implicated in the pathogenesis and progression of MDD and stress-associated psychiatric disorders

Given the rapid growth and increasing fragmentation of the literature on MDD epigenetics, this narrative review aims to construct a comprehensive, integrated knowledge framework by synthesizing advances across five core epigenetic regulatory pathways. Peer-reviewed articles published between January 2000 and June 2026 were retrieved from PubMed, Web of Science and Scopus. The combined search terms included depression, epigenetics, DNA methylation, histone modification, non-coding RNA, RNA modification, chromatin remodeling and epigenetic clocks. Original research papers, systematic reviews and meta-analyses focusing on the above five regulatory axes were prioritized during literature screening. Unlike previous reviews that have often focused on a single mechanism or a limited set of modifications [9], our work systematically encompasses the full spectrum of epigenetic regulation, including DNA methylation, histone modifications, non-coding RNAs, RNA chemical modifications, and chromatin remodeling (Fig. 1), with particular emphasis on their functional crosstalk and spatiotemporal specificity across distinct brain regions and cell types. Through multi-dimensional integration, we address a critical scientific question, namely how diverse epigenetic marks act in concert to encode environmental stress information into lasting transcriptional reprogramming and behavioral phenotypic changes. We also highlight emerging research directions that have received less attention in prior reviews, such as epitranscriptomic mechanisms including m⁶A modification and chromatin-remodeling pathways. From a translational perspective, we systematically evaluate the potential utility of peripheral epigenetic biomarkers for auxiliary diagnosis and treatment response prediction, and analyze the current landscape and challenges in developing central nervous system-targeted epigenetic therapeutics. In summary, by integrating the regulatory networks and translational evidence across multiple epigenetic layers, this review aims to provide a solid theoretical foundation for precision diagnosis and individualized treatment of MDD, and to facilitate the effective translation of basic research findings into clinical practice.

## Core epigenetic regulatory mechanisms underlying the pathogenesis of MDD

### DNA methylation dysregulation in MDD

DNA methylation tightly regulates gene transcription by covalently adding a methyl group to the 5′ carbon of cytosine residues, most commonly at CpG dinucleotides, to form 5-methylcytosine (5mC) [10]. This process is dynamically controlled through three key events: de novo methylation, during which DNA methyltransferases *DNMT3A* and *DNMT3B* establish methylation patterns at unmethylated CpG sites; maintenance methylation, in which *DNMT1* stably inherits methylation profiles during cell division; and demethylation. Active demethylation is mediated by the TET enzyme family through oxidizing 5mC, while passive demethylation arises from reduced inheritance of methylation marks due to impaired DNMT1 activity [11–14]. This balanced regulation sustains gene expression plasticity during development and environmental adaptation. When disrupted, it contributes closely to the onset of psychiatric disorders including MDD (Table 1.**)**.

**Table 1:** Aberrant DNA methylation of key target genes in major depressive disorder

Target Gene (Region)	Species	Tissue	Methylation Change	Clinical/Biological Outcome	References
*BDNF*(promoter I/IV)	Human、 rodent	Peripheral blood、 post-mortem brain, hippocampus, amygdala	Hypermethylation↑	Depression risk↑, cognitive impairment ↑, suicide risk↑; Asian-specific correlation; prenatal/chronic stress → *DNMT*↑ → *BDNF*↓ → neuronal development impairment	[15–21]
*NR3C1* (exon 1 F)	Human	Brain、peripheral blood、cord blood	Hypermethylation↑	HPA axis dysfunction ↑, stress response ↑; maternal depression during pregnancy → cord blood hypermethylation → intergenerational depression susceptibility	[22–24]
*SLC6A4* (promoter)	Human	Peripheral 、bloodlymphocyte cell line	Hypermethylation↑	Depression severity ↑, antidepressant response ↓, brain structural abnormality ↑; sex-specific methylation pattern; 5-HTTLPR s genotype amplifies adverse effects	[25–28]
*Bicd2*	Human 、Mouse	Peripheral blood, hippocampus	Blood hypermethylation ↑/ Hippocampus hypomethylation ↓	BDNF signaling dysfunction → depressive-like behaviors ↑; hippocampal *Bicd2* knockdown → *BDNF* ↑ → antidepressant effects	[29, 30]
*KLK8* (promoter)	Human	Peripheral blood	Aberrant methylation	Depressive symptom severity ↑	[31]
*FKBP5* (intron 7)	Human	Peripheral blood	Hypomethylation↓	Childhood adversity exposure → HPA axis dysregulation ↑; cooperates with *NR3C1* → intergenerational depression risk ↑	[32]
*COMT*	Human	Peripheral blood	Hypomethylation↓	Catecholamine levels ↓, depression-related pathophysiology ↑	[33, 34]

(↑=upregulate; ↓=downregulate; →=lead to)

#### Dysregulation of BDNF (Brain-Derived Neurotrophic Factor) methylation in MDD

**Fig. 2:**
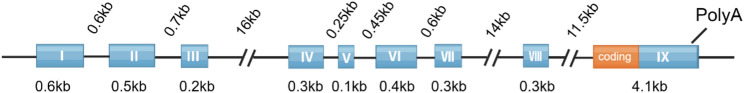
*Brain-derived neurotrophic factor* (*BDNF*) gene structure. Blue boxes represent exons, the black lines represent introns and the orange box indicates a coding exon

*BDNF* maintains central nervous system homeostasis by regulating neuronal survival, synaptogenesis, and synaptic plasticity. The *BDNF* gene contains nine exons (I–IX) controlled by independent promoters (Fig. 2), which provide multiple key sites for epigenetic modification [35]. Growing evidence links *BDNF* methylation to neurocognitive deficits in patients with MDD. Ferrer et al. reported that methylation at *BDNF* promoter I mediates the association between two *BDNF* polymorphisms (rs908867 and rs925946) and neurocognitive function. These results suggest DNA methylation could represent a tentative intermediate correlate connecting genetic variants and clinical traits [15]. Kang et al. further demonstrated that hypermethylation of the *BDNF* promoter in MDD patients correlates with suicide attempts, suicidal thoughts during treatment, and poor clinical outcomes related to suicidal risk [16].

Aberrant BDNF methylation also exhibits distinct population-specific patterns in human peripheral biospecimens, alongside brain-region-specific epigenetic profiles observed in preclinical models. A meta-analysis by Zhu et al. showed that BDNF hypermethylation in peripheral samples correlates with elevated depression risk only in Asian populations [17]. In saliva samples from Japanese patients with MDD, hypermethylation was detected at specific CpG sites in *BDNF* promoter I, while methylation at exon I was reduced. In oral tissue from elderly MDD patients (≥ 65 years), methylation levels at *BDNF* promoters I and IV were significantly higher than those in age-matched healthy controls [18]. Preclinical animal studies further support these regulatory patterns. Increased methylation at the *rodent BDNF* exon IV region was found in the hippocampus and amygdala of rats with prenatal stress and in adolescent mice exposed to chronic unpredictable mild stress (CUMS). These changes were accompanied by reduced BDNF expression and elevated expression of *DNMT1* and *DNMT3A*. High DNMT1 and *DNMT3A* levels were linked to impaired neuronal development. *DNMT* inhibitors could reverse these epigenetic abnormalities and improve behavioral deficits in adulthood [19, 20]. Niknazar et al. reported higher *BDNF* exon IV methylation in the hippocampus of female than male rats after chronic stress, suggesting a possible sex-specific epigenetic mechanism in rodents, although its relevance to human female vulnerability to depression requires further validation [21].

#### *NR3C1* methylation and HPA axis dysfunction in MDD

The glucocorticoid receptor (GR), encoded by the *NR3C1* gene, serves as a core regulator of the hypothalamic-pituitary-adrenal (HPA) axis stress response. Exon 1 F in the *NR3C1* promoter contains 14 methylation sites, representing 77.8% of the gene’s 18 total methylation sites and forming a key region for epigenetic regulation [36]. McGowan et al. showed that hypermethylation of the *NR3C1* promoter strongly reduces GR expression. This change impairs negative feedback regulation of the HPA axis, leads to abnormal stress responses, and increases susceptibility to MDD [22].

In a study of intergenerational effects of the prenatal environment on *NR3C1* methylation, Oberlander et al. first reported that maternal depression during pregnancy increases *NR3C1* methylation in neonatal umbilical cord blood. This methylation change, in turn, was associated with elevated salivary cortisol stress responses in infants at 3 months of age [36]. A study by Wei et al. during the COVID-19 lockdown further showed that pregnant women with MDD had significantly higher methylation at the *NR3C1* CpG8 site in peripheral blood mononuclear cells than healthy pregnant women. More severe depressive symptoms were associated with higher methylation at this locus [23]. These findings suggest that NR3C1 methylation may serve as a plausible intermediate pathway via which prenatal environmental factors correlate with intergenerational mental health outcomes. Studies in adult MDD patients have shown that individuals undergoing the Trier Social Stress Test (TSST) exhibit significantly blunted cortisol reactivity accompanied by increased global *NR3C1* methylation. The most pronounced changes appear at CpG12 and CpG20, which overlap with the NGFI-A binding site [24]. This finding expands the scope of *NR3C1* methylation research from early developmental stages to acute stress responses in adult pathological conditions.

#### SLC6A4 methylation and serotonin system dysregulation in MDD

*SLC6A4* encodes the serotonin transporter (5-HTT), a critical regulator of serotonin reuptake in the synaptic cleft. Its expression is synergistically modulated by 5-HTTLPR polymorphism and promoter methylation status. Yéssica et al. demonstrated that the 5-HTTLPR short allele is a significant risk factor for depression onset and severity [37], and that methylation can further amplify this genetic effect: in human adult lymphocyte cell lines, *SLC6A4* promoter hypermethylation is significantly associated with reduced mRNA levels, and both full and partial methylation markedly inhibit *SLC6A4* expression [25, 26].

Peripheral blood analyses showed that *SLC6A4* methylation at centric-to-terminal CpG sites (factor 1) was higher in women than men but unrelated to MDD severity in either sex; methylation at anterior-to-centric sites (factor 2), by contrast, scaled with MDD severity in women alone. These findings suggest sex-specific topography of *SLC6A4* methylation and its divergent ties to illness severity [27]. In patients with MDD, 5‑HTT mRNA levels in peripheral blood increased over 32 weeks of antidepressant treatment, and this increase was positively associated with improvement in HDRS scores. However, patients carrying the 5‑HTTLPR long allele or a higher proportion of the *STin2.12* allele exhibited poorer antidepressant responses [28].

#### Methylation abnormalities of other depression-susceptible genes

In addition to the core genes mentioned above, aberrant methylation of several novel susceptibility genes has been increasingly associated with MDD. In rodent models, *Bicd2* contributes to neural function by modulating the subcellular localization of BDNF and local mRNA translation [29]. In the CUMS mouse model of depression, *Bicd2* exhibits hypermethylation in peripheral blood but hypomethylation in the hippocampus. Furthermore, hippocampal *Bicd2* knockdown elicits antidepressant effects by upregulating *BDNF* expression [30].

Peripheral blood methylomic profiling of clinically diagnosed MDD patients identifies robust associations between methylation at seven distinct CpG sites within the *KLK8* promoter and the overall severity of depressive symptoms [31]. As a key regulatory cofactor of the HPA axis, FKBP5 exhibits hypomethylation in intron 7 in peripheral blood among children exposed to maternal depression, with significant concordance of methylation patterns between mothers and offspring. This suggests that *FKBP5* may act in concert with *NR3C1* to mediate the intergenerational transmission of MDD [32]. Goldenthal et al. found positive peripheral HTR1A − 681 CpG methylation-orbitofrontal gray matter correlations in 50 medication-naive MDD patients, verifying 5-HT₁A signaling’s neurotrophic effects. Childhood abuse altered gray matter across distinct brain regions but showed no association with HTR1A methylation (*p* > 0.05), indicating early trauma and abnormal HTR1A epigenetics independently induce depressive structural brain remodeling centered on the orbitofrontal cortex [38]. Moreover, hypomethylation at HCG9 (cg26892761) and CTDP1 (cg25300757) loci in patients with severe mental disorders (linked to depression via placental cis-mQTLs), as well as altered catecholamine levels associated with *COMT* hypomethylation, are all associated with depressive symptoms. These findings collectively provide novel insights into the epigenetic basis of MDD [33, 34, 39].

#### From candidate genes to EWAS: insights from genome-wide findings

Beyond the candidate-gene approach detailed above, epigenome-wide association studies (EWAS) offer an unbiased, hypothesis-free screening of the entire methylome, enabling discovery of novel MDD-associated loci. A Korean EWAS (350 MDD, 161 controls) identified 2,018 differentially methylated probes and 351 regions, with eight CpGs annotated to *EML6*, *ZFP64*, *CLSTN3*, *KCNMA1*, *TAOK2*, and *NT5E* correlating negatively with left ventrolateral prefrontal cortical thickness [40]. An EWAS of electroconvulsive therapy response identified DMRs annotated to *MIR596*, *MPIG6B*, *NRP2*, and *CYP1A1*, partially replicated across cohorts [41]. A Psychiatric Genomics Consortium methylome-wide association study (24,754 European participants, 5,443 cases; 243 East Asian cases, 1,846 controls) identified 15 CpGs associated with MDD, with effect sizes correlated across ancestries (*r* = 0.48). Notably, overlap between EWAS findings and candidate-gene associations is limited; even for *BDNF* and *SLC6A4*, EWAS have failed to replicate targeted associations. Poor cross-cohort replication of candidate-CpG associations, attributed to tissue type, sample composition, methodology, and population stratification, further underscores this disconnect [42]. These findings highlight the complementary roles of targeted and genome-wide strategies for understanding DNA methylation dysregulation in MDD.

#### Developmental windows and epigenetic programming by early-life adversity

A developmental sensitive window is a critical early-life stage during which the brain is highly sensitive to environmental signals. Stress exposure during this window can permanently change gene expression through epigenetic mechanisms and increase vulnerability to MDD in adulthood. This effect is central to the development of MDD. In Long-Evans rats, Weaver et al. demonstrated that differences in maternal licking and grooming (LG) stably program offspring HPA stress phenotypes. Adult offspring of low-LG mothers showed hypermethylation at the NGFI-A binding site in the hippocampal GR promoter and exhibited exaggerated adrenocorticotropic hormone and corticosterone HPA responses to acute stress [43].

Human studies further substantiate the importance of developmental sensitive windows. Analysis of postmortem brain tissue by McGowan et al. demonstrated that suicide victims with childhood abuse exhibited significantly decreased *GR* expression in the hippocampus and markedly elevated cytosine methylation within the *GR* promoter exon 1 F region. These alterations were absent in suicide victims without childhood abuse and in non-suicide control subjects [22]. Further studies indicate that individuals exposed to childhood abuse before 3 years of age exhibit more severe epigenetic aberrations in *BDNF* and *NR3C1* methylation in peripheral blood in adulthood. This suggests that the period before age 3 constitutes a critical window for DNA methylation sensitivity to early-life adversity [44].

### Abnormal regulation of histone modifications in major depressive disorder

Histone modifications govern gene expression in core pathways such as neuroplasticity and stress responses by dynamically remodeling chromatin conformation. Dysregulation of these marks is closely implicated in the pathogenesis and progression of MDD. The nucleosome is the basic structural unit of eukaryotic chromatin, consisting of a 147-bp DNA fragment wrapped around a histone octamer comprising two copies each of H2A, H2B, H3, and H4. The N-terminal tails of histones undergo diverse post-translational modifications, including acetylation, methylation, phosphorylation, and crotonylation, enabling precise control of gene transcription [45].

#### Histone acetylation imbalance and HDAC regulation

The dynamic equilibrium of histone acetylation is maintained by histone acetyltransferases (HATs) and histone deacetylases (HDACs). HATs catalyze the addition of acetyl groups to histone lysine residues, neutralizing positive charges to relax chromatin structure and promote transcription factor binding. In contrast, HDACs condense chromatin via deacetylation, thereby repressing gene transcription [46].Based on structural and functional characteristics, the HDAC family is classified into four classes: Class I (HDAC1/2/3/8) is nuclear-localized and primarily mediates epigenetic transcriptional regulation; Class II (HDAC4/5/6/7/9/10) shuttles between the nucleus and cytoplasm and deacetylates both histone and non-histone substrates; Class III (sirtuins) depends on NAD + for catalytic activity and coordinates metabolic and transcriptional regulation; Class IV comprises only HDAC11 [47].

Preclinical studies have demonstrated that chronic stress exposure induces region‑ and substrate‑specific alterations in histone acetylation. Kenworthy et al. reported that mice exposed to chronic social defeat stress (CSDS) exhibited significantly decreased acetylation at H3K9, H3K14, H4K5, and H4K8 in the dorsomedial nucleus and hippocampus. Conversely, upregulated *HDAC2* expression in the nucleus accumbens exacerbated depression-like behaviors in mice [48, 49]. Studies using the CUMS paradigm further demonstrate the brain regional specificity and complexity of histone acetylation dysregulation. In the hypothalamus, hippocampus, and parietal cortex of CUMS rats, acetylation levels (including H3K9ac and H4K8ac) and HDAC2/3/4/5 expression are significantly associated with susceptibility or resilience to behavioral phenotypes such as sucrose preference and object recognition [50]. Furthermore, Fuchikami et al. demonstrated that decreased H3 acetylation at the P1, P4, and P6 regions of the mouse hippocampal *BDNF* promoter suppresses *BDNF* transcription, resulting in downregulated expression [51]. In a rat model of Gulf War Syndrome‑associated depressive‑like behaviours, preclinical evidence has shown that ketamine increases acetylation at *BDNF* promoter IV by inhibiting HDAC activity, thereby upregulating *BDNF* expression, promoting hippocampal dendritic spine remodeling, and ultimately reversing synaptic plasticity deficits [52].

In parallel with these preclinical observations, clinical evidence from human samples has begun to implicate histone acetylation dysregulation in MDD. In a cohort of 56 patients with moderate‑to‑severe MDD, Cortés‑Erice et al. found that *HDAC5* and *SIRT2* exhibited nuclear enrichment in peripheral blood monocytes and T‑cells, with the *HDAC*5 cytoplasm/nucleus ratio negatively correlating with illness severity. *HDAC5* mRNA was upregulated in T‑cells and intermediate monocytes, while *BDNF* mRNA was decreased in these cells, suggesting a potential link between histone deacetylation and reduced neuroplasticity [53]. Critically, these peripheral immune molecular signatures lack functional validation within the human central nervous system and cannot be directly translated to interpret brain-specific epigenetic remodelling in MDD patients. Supplementary post-mortem brain analyses from suicide decedents further corroborate this epigenetic regulatory cascade: Misztak et al. documented significant H3K9/14ac depletion in the frontal cortex and hippocampus of suicide cases, concurrent with elevated HDAC3 protein and lowered BDNF protein concentrations [54].

#### Histone methylation dynamics

Histone methylation occurs predominantly at lysine (K) and arginine (R) residues on histones H3 and H4. Its function in transcriptional regulation is determined by the modification site and methylation status, including monomethylation (me1), dimethylation (me2), and trimethylation (me3). Among these marks, H3K4me3 and H3K36me3 are associated with transcriptional activation, whereas H3K9me2 and H3K27me3 are linked to transcriptional repression. This specificity is tightly regulated by lysine methyltransferases (KMTs) and lysine demethylases (KDMs) [55].

In preclinical models, In the nucleus accumbens (NAc) of mice susceptible to early-life stress (ELS) or chronic social defeat stress (CSDS), SUZ12 assembles functional PRC2 complexes via its VEFS domain, leading to aberrant elevation of H3K27me1. This epigenetic change has been linked to lifelong stress vulnerability by persistently altering gene expression and neuronal excitability [56]. Further studies have shown that mice exposed to ELS also exhibit altered adult stress responses via dynamic regulation of H3K79me2 in D2-type medium spiny neurons within the NAc. Systemic treatment with DOT1L inhibitors reverses ELS-induced behavioral impairments. In the ventral striatum of CUMS-exposed mice, reduced H3K4me3 occupancy at the GDNF promoter was accompanied by decreased GDNF transcription. Administration of HDAC inhibitors attenuated both the behavioural abnormalities and the suppression of GDNF expression, suggesting that histone modifications may contribute to stress-induced behavioural changes, at least in part, through the modulation of neurotrophic factor signalling [48]. Furthermore, in a mouse model of postnatal immune activation, high-intensity interval training (HIIT) during adolescence activates the histone demethylase KDM6B (which removes H3K27me3) in the prefrontal cortex, upregulates IL-4 and BDNF expression, attenuates neuroinflammation, and ameliorates adult-onset depression-like behavior [57].

Clinical studies have demonstrated that in peripheral blood mononuclear cells (PBMCs) from patients with MDD, H3K4me3 enrichment at the promoter of TNIP2 (a key mediator in the TLR4 signaling pathway) is significantly decreased and correlates negatively with depression severity [58]. Furthermore, Cruceanu et al. reported that in postmortem prefrontal cortex tissue from 18 suicide victims diagnosed with MDD, H3K4me3 enrichment at the *SYN1* promoter was associated with an altered expression balance between the neural circuit-related *SYN1a* and *SYN1b* isoforms; notably, this observation is limited to postmortem suicide MDD cases and cannot be generalized to all individuals living with MDD [59].

#### Dysregulation of histone phosphorylation and the novel modification crotonylation

Histone phosphorylation contributes to the rapid regulation of stress responses by altering chromatin charge status. This modification occurs predominantly at serine (Ser) and threonine (Thr) residues and is maintained by a dynamic balance between protein kinases (PKs) and protein phosphatases (PPs) [60]. Studies have demonstrated that H3S10 phosphorylation (H3S10p) is upregulated in the hippocampus of rodents mice subjected to fear conditioning or drug addiction models. This change has been linked to depression‑like behaviors, potentially by regulating the transcription of memory‑associated genes [61].

Histone crotonylation is dynamically regulated by crotonyltransferases and decrotonyltransferases [62]. In the chronic social defeat stress (CSDS) mouse model, histone crotonylation levels are decreased, accompanied by upregulated expression of chromodomain Y-like protein (CDYL) [63]. CDYL-mediated repression of VGF transcription in the prelimbic cortex is associated with reduced expression of synaptic plasticity-related proteins. It is therefore plausible that histone crotonylation dysregulation may affect synaptic integrity and thereby contribute to MDD pathophysiology, although the causal relationship remains to be established [64].

#### Stress-dependent dynamic features of histone modifications

The high dynamism of histone modifications represents a hallmark of the stress response; their half-life is only a few minutes in transcriptionally active chromatin [65].

In preclinical models, histone acetylation and methylation dynamics have been shown to participate in stress‑induced behavioral adaptations. In the chronic social defeat stress (CSDS) mouse model, H3K14 acetylation levels in the NAc transiently decrease 1 h after stress, increase at 24 h, and persist for 10 days. These changes exhibit a significant negative correlation with elevated *HDAC2* expression. Infusion of the Class I HDAC inhibitor MS-275 into the NAc reverses aberrant H3K14ac modifications and ameliorates depressive-like behaviors (including sucrose preference loss and social avoidance) in mice [66]. A study using the novel Progressive Social Defeat Stress (PSDS) paradigm demonstrated that during stress-induced progression of depressive phenotypes, the hippocampus and dentate gyrus (DG) exhibit time- and region-specific methylation dynamics. H3K9me2 is persistently downregulated after day 4 of stress, whereas H3K27me2 is initially upregulated in the DG and then downregulated after day 5. These changes are accompanied by early upregulation of the demethylase genes *PHF8* and *PHF2*. These dynamic alterations coincide with the onset and progression of depressive-like behaviors [67]. Similar chromatin signatures, including increased H3K14 acetylation and reduced *HDAC2* expression, have been observed in postmortem nucleus accumbens tissue from patients with MDD, paralleling the changes found in stress‑susceptible mice [68].

### Regulatory roles of non-coding RNAs in major depressive disorder

**Fig. 3:**
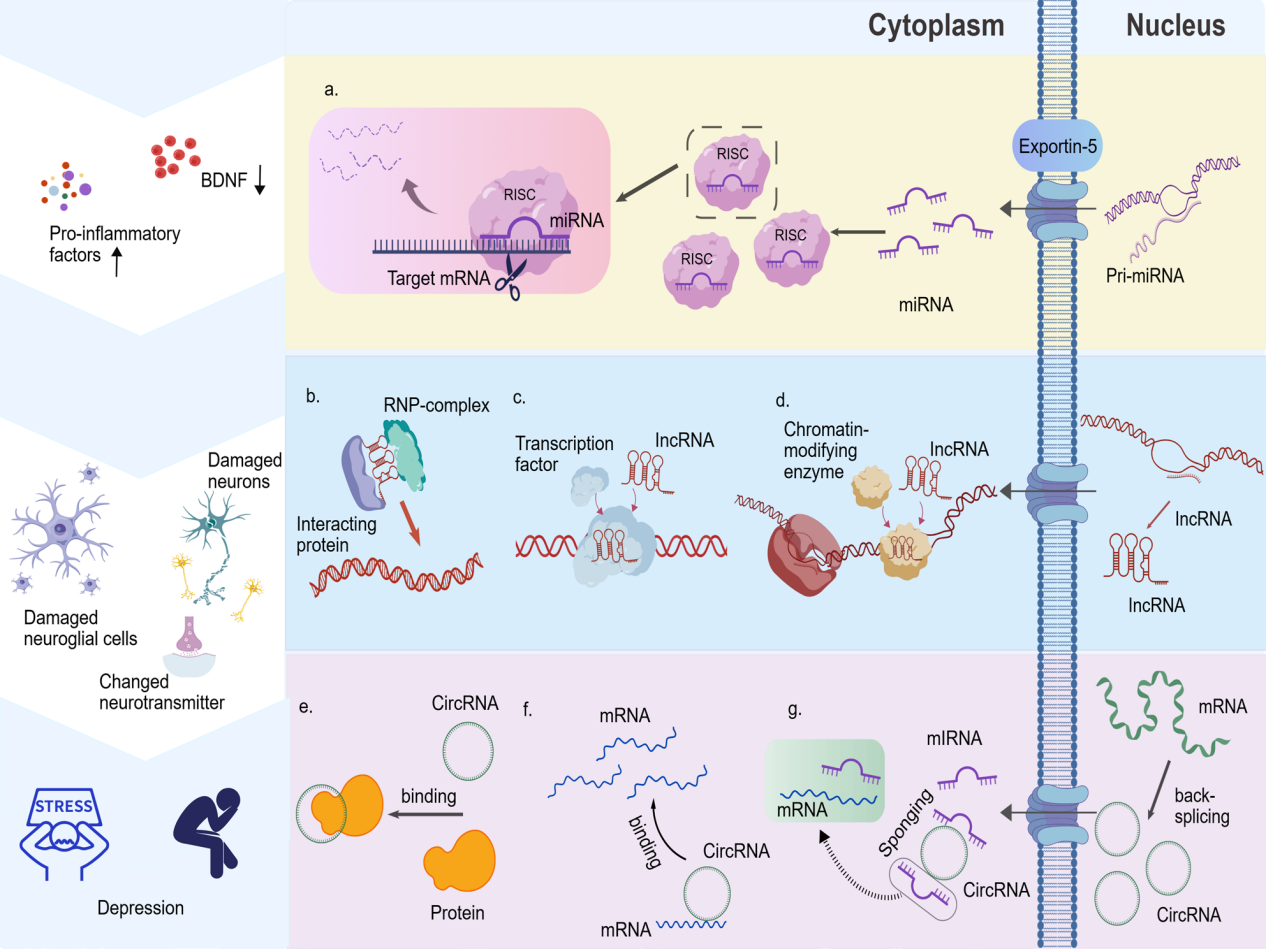
Schematic diagram of non-coding RNA-mediated epigenetic regulatory mechanisms in MDD. This illustration depicts the biogenesis and regulatory modes of three classes of ncRNAs in MDD pathogenesis: **(a)** Biogenesis and post-transcriptional silencing of miRNAs: pri-miRNAs are processed and exported from the nucleus via Exportin-5, then cleaved and assembled into the RNA-induced silencing complex (RISC) in the cytoplasm. Mature miRNAs recognize target mRNAs to trigger degradation or translational repression; **(b)** lncRNAs act as scaffolds to recruit proteins and assemble ribonucleoprotein (RNP) complexes; **(c)** lncRNAs modulate transcription by directly interacting with transcription factors; **(d)** lncRNAs recruit chromatin-modifying enzymes to reshape chromatin structure; **(e)** circRNAs bind to target proteins to modulate their activity and localization; **(f)** circRNAs interact with mRNAs to regulate stability and translational; **(g)** circRNAs function as miRNA sponges to relieve their post-transcriptional repression of target mRNAs.Chronic stress promotes the pathogenesis of MDD by inducing neuroinflammation, reduce *BDNF* expression, neuronal damage, glial cell function, and neurotransmitterimbalance. Dysregulation of the aforementioned ncRNA pathways serves as a critical epigenetic mediator that connects environmental stressors to the pathological alterations underlying MDD

Non-coding RNAs (ncRNAs) generally lack the ability to encode canonical full-length proteins and function to fine-tune gene expression via transcriptional and post-transcriptional regulatory mechanisms, while a small fraction of lncRNAs and circRNAs carry short open reading frames (sORFs) to produce functional micropeptides [69]. Based on molecular length and structural characteristics, ncRNAs are mainly classified into three categories: microRNAs (miRNAs), long non-coding RNAs (lncRNAs), and circular RNAs (circRNAs). In the pathophysiology of MDD, aberrant ncRNA expression profiles are closely associated with impaired neuroplasticity, dysregulated stress responses, and disrupted neurotransmitter systems. Importantly, antidepressant treatment can reverse these abnormal expression patterns [70] (Fig. 3).

#### Regulatory mechanisms of microRNAs in the pathogenesis of MDD

MicroRNAs (miRNAs) are endogenous small non-coding RNAs (ncRNAs) approximately 18–25 nucleotides (nt) in length. Their biosynthesis involves two-step processing in the nucleus and cytoplasm: first, primary miRNAs (pri-miRNAs) are transcribed by RNA polymerase II and cleaved by the Drosha-DGCR8 complex into precursor miRNAs (pre-miRNAs). Subsequently, pre-miRNAs are exported to the cytoplasm by Exportin-5 and cleaved by Dicer into mature miRNAs [71]. Mature miRNAs bind to the 3′ untranslated region (3′UTR) of target mRNAs, triggering mRNA degradation or translational repression. In this way, miRNAs exert key regulatory roles in neurogenesis, synaptic plasticity, and stress responses [72, 73].

In patients with MDD and animal models of depression, miRNA dysregulation is predominantly implicated in pathways related to neuroplasticity, neurotransmitter systems, and stress responses.Regarding neuroplasticity regulation, hippocampal overexpression of miR-206-3p in mice induces depressive-like behaviors by suppressing *BDNF* signaling and reducing the number of doublecortin-positive (DCX-positive) cells. Inhibition of miR-206-3p reverses these effects, enhancing hippocampal neurogenesis and alleviating depressive symptoms [74]. Furthermore, miR-15, miR-182, and miR-206 are significantly upregulated in the peripheral blood and postmortem brain tissue of patients with MDD. These miRNAs directly target and repress *BDNF* expression, resulting in impaired neuronal survival and synaptogenesis, thereby contributing to MDD pathogenesis [75]. Dysregulation of neurotransmitter system-associated miRNAs is equally critical, and synergistic regulatory networks exist in which multiple miRNAs can target the same gene. For instance, miR-17 and miR-92 (which target *SLC6A4*) are upregulated in the peripheral blood of in patients with MDD. By downregulating *SLC6A4* expression, these miRNAs disrupt serotonin reuptake in the synaptic cleft. Conversely, reduced miR-4775 expression may help maintain *SLC6A4* function through a positive regulatory mechanism. In stress response pathways, miRNAs contribute to MDD pathogenesis by modulating inflammatory and cell survival signaling. miR-27a is downregulated in the serum and hippocampus of patients with MDD. In primary microglia, isoliquiritigenin exerts antidepressant effects by upregulating miR-27a, which specifically represses spleen tyrosine kinase (Syk) and the downstream NF-kB pathway, thereby attenuating *NLRP3* inflammasome-mediated pyroptosis [76].

#### Long non-coding RNA expression and function

Long non-coding RNAs (lncRNAs) are RNA transcripts longer than 200 nucleotides (nt), which can be classified by their genomic localization into intergenic, antisense, intronic and other subtypes [77]. Their regulatory functions exhibit distinct nuclear-cytoplasmic compartmentalization. In the nucleus, lncRNAs modulate gene transcription by mediating chromatin remodeling, recruiting transcription factors, or acting as enhancers. In the cytoplasm, lncRNAs function as miRNA sponges, regulate mRNA stability, or promote protein complex assembly, thereby achieving multidimensional regulation of gene expression [78].

Studies have demonstrated that peripheral blood from patients with MDD shows extensive aberrant expression of lncRNAs and mRNAs. Differentially expressed mRNAs are mainly enriched in pathways related to basal metabolism and neurodevelopment. Co-expression network analysis further identified key lncRNAs including chr10:874695–874,794 (human reference genome assembly hg19/GRCh37), which may contribute to MDD pathogenesis by modulating downstream mRNA expression [79]. In addition, lncRNA expression is downregulated in peripheral blood mononuclear cells (PBMCs) from patients with MDD, and these expression levels are negatively correlated with suicide risk [80]. Aberrant lncRNA expression also displays brain region-specific and sex-specific patterns.

For instance, the primate-specific lncRNA *LINC00473* is downregulated in the prefrontal cortex of female patients with MDD, whereas no significant changes are observed in male patients. In mouse models, overexpression of *LINC00473* in the medial prefrontal cortex was associated with increased stress resilience selectively in females, accompanied by sex-specific alterations in synaptic function and gene expression [81].

#### Regulatory networks and biological roles of circular RNAs in MDD

Circular RNAs (circRNAs) are covalently closed-loop RNA molecules generated by back-splicing of precursor messenger RNAs (pre-mRNAs), characterized by high structural stability and pronounced tissue specificity [82]. Their core biological function is to act as endogenous miRNA sponges, thereby alleviating the post-transcriptional inhibitory effects of miRNAs on target mRNAs. In addition, circRNAs can regulate gene expression by interacting with functional proteins or encoding small peptides via translation [83].

In preclinical models, circRNAs have emerged as regulators of depression‑related phenotypes, with evidence pointing to their involvement in neurogenesis and inflammatory processes. Regarding neurogenesis regulation, *circANKS1B* is significantly downregulated in the hippocampal dentate gyrus (DG) of CUMS rats. Its binding capacity for miR-146a-5p is impaired, leading to enhanced repression of its target gene *KLF4* by miR-146a-5p and subsequent suppression of hippocampal neurogenesis. Conversely, overexpression of *circANKS1B* restores *KLF4* expression, promotes hippocampal neurogenesis, and ameliorates depressive-like behaviors in rats [84]. In a related line of investigation, *circDYM* binds the transcription factor *TAF1*, an interaction associated with suppressed expression of TAF1‑dependent pro‑inflammatory genes. In CUS‑exposed mice, *circDYM*‑enriched extracellular vesicle administration correlated with reduced microglial activation, limited peripheral immune infiltration, preserved BBB integrity, and improved depression‑like behaviours. Together, these findings suggest a role for *circDYM* in stress‑related neuroinflammation [85]. Consistent with the preclinical circRNA expression patterns, parallel alterations of circular RNAs have also been detected in peripheral blood samples from patients with MDD. *CircFKBP8* and *circMBNL1* display aberrant expression in the whole blood of MDD patients, with circFKBP8 downregulated and *circMBNL1* upregulated; both can only be regarded as tentative candidate peripheral biomarkers at present. Correlative analyses indicate *circMBNL1* may participate in the regulation of neuroplasticity via modulating peripheral *BDNF* concentrations, yet direct causal evidence linking peripheral *circMBNL1* alterations to central nervous system neuroplastic remodeling remains lacking [86]. Mirroring the expression trend observed in preclinical animal models, circulating *circDYM* concentrations are significantly reduced in the plasma of patients with major depressive disorder, and this change correlates inversely with systemic inflammatory markers.

### Roles of RNA epigenetic modifications in MDD

RNA modification represents a core regulatory mechanism in epigenetic transcriptomics. By dynamically attaching chemical moieties (e.g., methyl or acetyl groups) to RNA molecules (including mRNAs, tRNAs, and rRNAs), RNA modifications regulate RNA splicing, nucleocytoplasmic transport, translational efficiency, and degradation kinetics without changing the underlying nucleic acid sequence, thus shaping the spatiotemporal specificity of gene expression [87]. In the central nervous system (CNS), RNA modifications are essential for the precise regulation of neurogenesis, synaptic plasticity, and stress responses. Dysregulation of these modifications is closely implicated in the pathophysiology of neuropsychiatric disorders, including MDD.

#### m⁶A modification and its regulatory mechanisms

The regulation of N⁶-methyladenosine (m⁶A) modification relies on a three-component regulatory network composed of “writers, erasers, and readers”: writers (methyltransferase complexes) catalyze m⁶A formation; erasers (demethylases) remove methyl groups; and readers (binding proteins) recognize m⁶A-modified sites and mediate subsequent RNA metabolic processes. The core catalytic subunits of m⁶A writers predominantly include methyltransferase-like 3 *(METTL3)*, methyltransferase-like 14 *(METTL14)*, Wilms tumor-associated protein 1 *(WTAP)*, and RNA-binding motif protein 15 *(RBM15)*.Among these, *METTL3* serves as the catalytically active core subunit, which uses S-adenosylmethionine (SAM) as a methyl donor to transfer methyl groups to the sixth nitrogen atom of adenine residues in mRNAs [88].

In preclinical models, in the CUMS rat model, *METTL3* expression is significantly upregulated in the hippocampus. *METTL3* catalyzes m⁶A modification of primary microRNA (pri-miR-221), increasing the binding efficiency between DiGeorge syndrome critical region 8 (*DGCR8*) and pri-miR-221, thereby facilitating the processing of pri-miR-221 into functional miR-221-3p. This mature miRNA further targets and represses the expression of GRB2-associated binding protein 1 (*Gab1*), exacerbating CUMS-induced cognitive impairments and inducing depressive-like behaviors [89]. In neural stem cells (NSCs), specific knockdown of *METTL3/METTL14* reduces m⁶A modification levels, inhibits NSC proliferation, impairs their differentiation into neurons, and simultaneously promotes aberrant glial cell differentiation. Conversely, *METTL3* overexpression facilitates NSC differentiation into neurons. Mechanistically, *METTL3* enhances the stability of low-density lipoprotein receptor-related protein 2 (*Lrp2*) mRNA via m⁶A modification and increases its translational efficiency through the reader protein YTHDC2. As a neurotrophic factor receptor, *Lrp2* upregulation significantly promotes hippocampal neurogenesis. Animal studies further support that hippocampal-specific *METTL3* knockout mice exhibit reduced hippocampal neurogenesis, impaired spatial memory, and depressive-like behaviors, whereas hippocampal *Lrp2* overexpression reverses these behavioral deficits [90].

As a key m⁶A eraser, downregulated fat mass and obesity-associated protein (*FTO*) expression is a common feature in both patients with MDD and animal models of depression. Liu et al. found reduced *FTO* mRNA in human post-mortem hippocampal tissue and functional alterations linked to *FTO* expression in mouse models of depression. Hippocampus-specific knockdown or conditional knockout of *FTO* induces typical depressive-like behaviors in adult mice, while hippocampal *FTO* overexpression rescues depression-related behavioral deficits [91].

#### ac4C and m⁵C modifications in depression

In addition to N⁶-methyladenosine (m⁶A), other RNA modifications including N4-acetylcytidine (ac4C) and 5-methylcytosine (m⁵C) participate in the pathogenesis of major depressive disorder (MDD) through distinct regulatory mechanisms. The ac4C modification is catalyzed by N-acetyltransferase 10 (*NAT10*), which modulates mRNA translational efficiency by attaching an acetyl group to the N4 site of cytosine residues in mRNAs [92]. In hippocampal tissue from CUMS mouse models, *NAT10* expression is upregulated, which triggers anxiety-and depressive-like behavioral phenotypes in mice. Pharmacological inhibition of *NAT10* activity markedly ameliorates these anxiety-and depressive-like behaviors [93].

The m⁵C modification is mainly catalyzed by methyltransferases of the NSUN family (including *NSUN2* and *NSUN5*), which regulate protein synthesis by modulating translational accuracy and efficiency. As the major m⁵C methyltransferase in the CNS, *NSUN2* undergoes aberrant functional alterations that are closely linked to synaptic dysfunction underlying MDD. Studies have demonstrated that conditional knockout or overexpression of *Nsun2* in the mouse prefrontal cortex bidirectionally regulates depressive-like behaviors. *Nsun2* deficiency reduces tRNA m⁵C levels, leading to insufficient expression of glycine-decoding tRNAs (accounting for 70% of the total tRNA pool), specific translational efficiency impairment, and ultimately synaptic dysfunction and depressive-like behaviors [94].

### Chromatin remodeling complex dysregulation in depression

Chromatin remodeling dynamically regulates the position, structure and composition of nucleosomes via ATP-dependent chromatin-remodeling complexes, thereby altering genomic DNA accessibility and enabling precise control of gene transcription [95]. Based on structural and functional differences in their ATPase subunits, chromatin-remodeling complexes are mainly divided into four core families: the *SWI/SNF* family (centered on *BRG1/BRM* ATPases that drive chromatin decompaction), the ISWI family (including the ACF complex, which participates in nucleosome assembly and spacing regulation), the INO80 family (mediating nucleosome sliding and histone exchange), and the CHD family (which regulates transcription by modifying chromatin structure) [96]. In a preclinical rat model, integrative multi-omics profiling of fluoxetine across 27 brain regions revealed pervasive, region-specific chromatin remodelling and transcriptional changes, suggesting that dysregulation of chromatin-remodelling complexes may contribute to transcriptional imbalance in genes involved in neuroplasticity, stress responses, and energy metabolism, and that antidepressant treatment may partially restore gene expression homeostasis by reversing such changes [97].

Using a chronic stress mouse model and NAc samples from patients with MDD, Sun et al. demonstrated that chronic stress upregulates expression of the ISWI-family ACF complex. This complex represses gene expression by promoting nucleosome accumulation at the transcription start sites of stress-sensitive genes, thus blocking transcription factor binding. Functional experiments further supported that NAc-specific knockdown of *SNF2h* (a core subunit of the ACF complex) significantly reverses stress-induced abnormal nucleosome positioning, restores expression of stress-related genes, and ameliorates depressive-like behaviors in mice [98].

The *SWI/SNF* complex ATPase subunit *BRG1* (encoded by *SMARCA4*) plays a pivotal role in stress responses. In the social defeat stress mouse model, reduced nuclear translocation of *BRG1* in dopaminergic-innervated brain regions (including the NAc and striatum) decreases chromatin accessibility of immediate-early genes *c-Fos* and *Arc*. Further studies have shown that specific knockout of *Brg1* enhances stress resilience against social defeat and reduces cocaine-mediated rewarding effects in mice. This process is independent of dopaminergic neuronal activity; instead, it represses stress-associated aberrant transcription by increasing heterochromatin proportion and globally reducing chromatin accessibility.Furthermore, mice with congenital inactivation of *BRM* (encoded by *SMARCA2*), another SWI/SNF-family subunit, also display prominent stress resilience [99].

Members of the CHD family regulate the development of anxiety-depression comorbidity by regulating chromatin folding, and their functional dysregulation displays marked brain-region specificity. In high-anxiety-behaviour (HAB) mice, *CHD3* and *CHD5* protein expression was dysregulated in the ventral hippocampus in the absence of corresponding mRNA changes, pointing to post-transcriptional regulation. Given their roles in NuRD-like chromatin remodelling complexes, this protein-level imbalance has been linked to altered transcriptional programmes potentially relevant to anxiety-related phenotypes [100].

## Clinical translation of epigenetic research in depression

### Clinical value of epigenetic biomarkers

The clinical translation of epigenetic biomarkers for MDD holds considerable promise, yet a substantial gap remains between exploratory discovery and validated clinical application. Across diagnostic and predictive domains, emerging findings have linked various epigenetic measures to disease phenotypes and treatment outcomes. However, most evidence to date is preliminary in nature, with limited independent replication, standardized protocols, or prospective validation. Thus, the transition of these candidate markers into clinically actionable tools will require rigorous evaluation in large-scale, multi-center, and prospectively designed studies.

#### Epigenetic biomarkers for depression diagnosis

Accumulating findings derived from post-mortem human brain tissues, human peripheral biospecimens and animal models have identified multiple categories of epigenetic candidate biomarkers associated with MDD (Table 2.), encompassing gene-specific DNA methylation profiles, diverse non-coding RNAs, multi-omics-screened transcriptional signatures and genome-wide epigenetic clocks. While these epigenetic molecules exhibit tentative potential for MDD diagnosis and disease severity stratification, distinct tissue-specific expression disparities are observed between central brain tissue and peripheral blood. Moreover, the majority of these markers remain at the exploratory stage, requiring systematic validation in large, independent prospective cohorts before clinical implementation.

DNA methylation of the *BDNF* gene is the most widely studied epigenetic biomarker with diagnostic potential for MDD. Methylation levels at CpG sites in promoters I and IV are significantly increased in peripheral blood and postmortem brain tissue from MDD patients. These levels are negatively correlated with *BDNF* gene expression [101]. Similarly, hypermethylation of the *SLC6A4* promoter has been investigated as a potential diagnostic biomarker in specific clinical contexts, namely post‑stroke depression (PSD). In a longitudinal cohort study of 286 patients assessed at 2 weeks and 1 year after stroke, higher *SLC6A4* promoter methylation was independently associated with PSD at both time points and with worsening depressive symptoms over the follow‑up period. Notably, this association was observed only in patients carrying the 5‑HTTLPR s/s genotype, suggesting a gene‑by‑epigenetic interaction in PSD risk stratification [102]. For *Bicd2*, a multi‑cohort study in the Chinese Han population (*n* = 1,526 across four independent cohorts) reported DNA hypermethylation and mRNA downregulation in peripheral blood of MDD patients. In a CUMS mouse model, *Bicd2* showed hypomethylation and upregulation in the hippocampus, a region‑specific pattern that contrasts with the findings in blood. Hippocampal *Bicd2* knockdown exerted antidepressant‑like effects, an effect that was associated with activation of the BDNF/TrkB/Akt pathway. These cross-species observations suggest that *Bicd2* may serve as a candidate biomarker with potential mechanistic relevance, although the relationship between peripheral and central methylation changes warrants further investigation [30]. Notably, the opposite direction of *Bicd2* methylation change between peripheral blood (hypermethylation) and hippocampus (hypomethylation) in the same CUMS model underscores the limited cross-tissue concordance and cautions against using peripheral *Bicd2* methylation as a direct proxy for central epigenetic events.

Several ncRNAs have been investigated as peripheral blood biomarkers for MDD. *circMBNL1* is significantly upregulated in whole blood from patients with MDD and produces antidepressant effects by regulating *BDNF* levels. Functional magnetic resonance imaging (fMRI) analyses reveal correlative links between altered *circMBNL1* levels and disrupted spontaneous neural activity in the prefrontal cortex. Such correlated molecular and neuroimaging readouts suggest *circMBNL1* could serve as a tentative peripheral biomarker for MDD, while offering indirect insights into central nervous system functional disturbances and potentially broadening its translational prospects [86]. *circSTAG1* expression was found to be significantly decreased in the peripheral blood of patients with MDD, with its levels negatively correlated with disease severity. Mechanistic investigations in cellular and mouse models have provided evidence that *circSTAG1* overexpression may sequester the m⁶A demethylase *ALKBH5* in the cytoplasm, thereby reducing its nuclear translocation. This sequestration was associated with increased m⁶A modification and subsequent degradation of FAAH mRNA, which coincided with preserved astrocyte function and amelioration of depressive-like behaviours [103]. These findings collectively position *circSTAG1* as a candidate peripheral indicator and a potential target for further mechanistic inquiry, though human validation is still needed. For lncRNAs, genetic polymorphisms of *LINC01108* and *LINC00998* in peripheral blood of MDD patients have been associated with the expression levels of these lncRNAs and may influence susceptibility to depression [104]. In animal models, the lncRNA *TCONS_00019174* is downregulated in the hippocampus and has been shown to reverse depression‑like behaviors by activating the Wnt/β‑catenin signaling pathway; its expression level has also been linked to the therapeutic effect of imipramine, suggesting that it may serve as a candidate diagnostic marker, findings that await translation to human cohorts [105].

In addition to the aforementioned human association studies, multi‑omics and machine‑learning approaches have been applied to enhance biomarker discovery. Leveraging peripheral blood transcriptomic profiles from the training cohort GSE98793 and two independent external validation datasets including GSE76826 and GSE201332, investigators integrated weighted gene co-expression network analysis (WGCNA) with three machine learning algorithms, namely Random Forest, SVM-RFE and LASSO, to identify five putative diagnostic gene candidates: *S100A12*, *TIGIT*, *SERPINB2*, *GRB10* and *LHFPL2*. Among these markers, the neuroinflammation-associated calcium-binding protein *S100A12* exhibited the most robust diagnostic capacity, with an AUC of 0.89, 83.7% sensitivity and 85.2%. Nonetheless, these transcripts remain preliminary biomarker candidates that require validation in expanded independent clinical cohorts, and the inflammation-centric gene signature uncovered herein hints at a potential pathogenic link between perturbed inflammatory signaling and major depressive disorder [106].

In addition to locus-specific methylation biomarkers, DNA methylation-based epigenetic clocks have emerged as a complementary, systems-level approach for assessing biological aging in MDD. These clocks, including the Horvath pan-tissue clock, Hannum, PhenoAge, and GrimAge, estimate biological age from genome-wide DNA methylation patterns and have been shown to predict morbidity and mortality beyond chronological age across diverse populations [107–109]. Multiple independent studies have reported accelerated epigenetic aging in MDD. In a cohort of 49 unmedicated patients with MDD and 60 healthy controls, GrimAge acceleration was significantly greater in the MDD group, corresponding to a median of 2 years of excess cellular aging [110]. A subsequent study in untreated MDD patients confirmed significant acceleration in HannumAge and GrimAge, along with decreased DNA methylation-based natural killer cell proportions [111]. Large-scale cross-sectional analyses further demonstrated that lifetime MDD was significantly associated with GrimAge-PAD and PhenoAge-PAD, with odds ratios ranging from 1.21 to 1.30 [112]. Beyond diagnostic status, epigenetic age acceleration has been linked to clinically meaningful outcomes: greater acceleration correlates with depression severity, cognitive deficits, earlier illness onset, and poorer antidepressant treatment response [113]. Collectively, these findings position epigenetic clocks as promising composite biomarkers that integrate multiple methylation changes into a single clinically interpretable metric, complementing gene-specific methylation markers for risk stratification and treatment monitoring in MDD. Nevertheless, prospective longitudinal studies are warranted to establish their temporal dynamics and clinical utility in routine practice.

**Table 2:** Stratified summary of epigenetic biomarkers for MDD diagnosis

BiomarkerCategory	Species	Biospecimen	Molecular Change	Core Signaling Path	Clinical Characteristics	References
Gene DNA methylation	Human	Peripheral blood, saliva, post-mortem prefrontal cortex	*BDNF* promoter I/IV methylation ↑	*BDNF* expression ↓	Methylation level negatively correlates with BDNF expression	[101]
	Human	Peripheral blood	*SLC6A4* promoter methylation ↑	5-HT transport ↓	Validated in 286-patient longitudinal cohort; predictive only in 5-HTTLPR s/s genotype patients with post-stroke depression	[102]
	Human, Mouse	Human peripheral blood, mouse hippocampus	Blood methylation ↑; hippocampus methylation ↓	BDNF/TrkB/Akt(after knockdown) ↑	Four independent Han Chinese cohorts(total *n* = 1,526);tissue-and species-specific methylation patterns	[30]
circRNA	Human	Whole blood	*circMBNL1* expression ↑	BDNF signaling ↓	Correlates with disrupted prefrontal spontaneous neural activity	[86]
circRNA	Human, Mouse, cell lines	Peripheral blood	*circSTAG1* expression ↓	m⁶A/*ALKBH5*/FAAH ↓	Negatively correlates with MDD severity; astrocyte protective effect validated in preclinical models	[103]
lncRNA	Human	Peripheral blood	*LINC01108*, *LINC00998* expression altered by gene polymorphism	Neuroplasticity ↓	Gene variants affect individual depression susceptibility	[104]
lncRNA	Mouse	Hippocampus	*TCONS_0001917*4 expression ↓	Wnt/β-catenin ↓	Preclinical animal evidence only; no human validation	[105]
Inflammatory transcript	Human	Peripheral blood	*S100A12*, *TIGIT*, *SERPINB2*, *GRB10*, *LHFPL2*: differential expression	Neuroinflammation ↑	Screened from GSE98793, GSE76826, GSE201332; AUC = 0.89, Se = 83.7%, Sp = 85.2% (*S100A12*); validation needed	[106]
Epigenetic clock panel	Human	Peripheral blood	Epigenetic age acceleration ↑	Biological aging (composite)	Linked to severity, cognitive impairment, earlier onset, and poorer treatment response	[107–113]

(↑=increase; ↓=decrease)

#### Epigenetic markers for predicting treatment response

Preclinical mechanistic studies have uncovered multiple epigenetic and ncRNA candidates implicated in antidepressant response (Table 3.), and subsequent clinical investigations have extended some of these findings to peripheral biomarkers with preliminary predictive utility. Nonetheless, most evidence remains confined to single-center or exploratory cohorts without independent replication, and the translational value of these markers as reliable predictors of therapeutic outcomes awaits confirmation in large, well-powered, and prospectively designed studies.

Preclinical mechanistic studies have provided insights into the molecular basis underlying treatment response prediction. For instance, *circDYM* has been shown to regulate microglial activation via sponging miR‑9 to upregulate *HECTD1* and promote HSP90 ubiquitination [106]. In rodent models of depression, restoration of hippocampal *circDYM* expression after antidepressant treatment has been associated with alleviation of depression‑like symptoms, suggesting that the functional status of this signaling pathway may be mechanistically linked to therapeutic outcomes [114].

Translating these mechanistic insights to clinical populations, several cohort studies have explored peripheral epigenetic and non‑coding RNA markers for predicting antidepressant responses, methylation levels at CpG sites in the promoter regions of *BDNF*, *NR3C1*, *FKBP5*, and *SLC6A4* were significantly reduced in peripheral blood. These epigenetic changes showed different predictive values for treatment outcome. Alterations in *FKBP5* methylation showed the strongest ability to predict therapeutic efficacy. Among these, changes in FKBP5 methylation showed the strongest association with therapeutic efficacy; notably, a reduction threshold of > 15% in FKBP5 methylation at week 4 was associated with a response rate of 82.1% at day 100, compared with 38.5% in those with less reduction [115]. In another study conducted over 32 weeks of antidepressant treatment, the upregulation magnitude of peripheral blood 5-HTT mRNA was significantly positively correlated with clinical symptom improvement. However, carriers of the 5-HTTLPR long allele or the *STin2.12* allele showed lower efficiency in reversing *SLC6A4* hypermethylation and poorer therapeutic responses. This finding indicates that genotype-epigenotype interactions may need to be considered in predicting individual therapeutic efficacy [28]. A separate study examining prenatal SSRI exposure found that children with in‑utero citalopram exposure exhibited no significant differences in peripheral blood methylation levels of stress-related genes (*NR3C1* and *BDNF*) compared with unexposed controls, they displayed more severe attention-deficit/hyperactivity disorder (ADHD) symptoms as well as communication and psychomotor developmental delays during school age. This observation suggest that prenatal antidepressant exposure may influence long‑term neurodevelopment through epigenetic mechanisms. These observations also highlight the importance of considering life‑stage‑specific physiological characteristics when applying methylation‑based biomarkers [116].

Circ RNAs have also been explored as candidate predictors of treatment response. circHIPK2 is upregulated in the plasma of patients with MDD in a preliminary study, with a reported diagnostic AUC of 0.796 (sensitivity = 60.4%, specificity = 97.9%). Notably, analysis revealed that patients with a ≥ 20% reduction in circHIPK2 expression at day 14 of treatment achieved an 8‑week therapeutic response rate of 78.3%, whereas those with a reduction of < 20% showed a 32.6% response rate. These findings suggest that *circHIPK2* may have potential utility for early prediction of SSRI efficacy [117]. In clinical samples, *circDYM* is also downregulated in the peripheral blood of patients with MDD, and its restoration after antidepressant treatment has been correlated with symptomatic improvement, consistent with the preclinical observations described above, However, it should be noted that sufficient data are still lacking to clarify the signaling crosstalk between circulating circDYM and brain tissue [114].

**Table 3:** Epigenetic biomarkers for predicting antidepressant therapeutic response

Biomarker Category	Species	Biospecimen	Molecular Change	Core Signaling Pathway	Clinical Characteristics	References
Gene -specific DNA methylation	Human	Peripheral blood	Post-treatment methylation ↓: *BDNF*, *NR3C1*, *FKBP5*, *SLC6A4*; 5-HTT mRNA ↑ after long-term treatment	HPA axis / 5-HT neurotransmission	(1) *FKBP5* methylation reduction>15% at week4 corresponds to 82.1% remission at day100 (vs. 38.5% for smaller reduction); (2) 5-HTT mRNA upregulation positively correlates with symptom improvement over 32 weeks; (3) Carriers of 5-HTTLPR long / *STin2.12* allele show impaired *SLC6A4* demethylation and inferior therapeutic response	[28, 115]
CircRNA	Human, Rodent	Peripheral blood, plasma	Baseline *circDYM* ↓; expression recovers after antidepressant treatment	miR-9/*HECTD1*/HSP90 axis regulating microglial activation	Lower baseline circDYM level predicts poorer symptom relief; preclinical rodent results are consistent with human clinical observations	[114]
CircRNA	Human	Plasma	Baseline *circHIPK2* ↑; expression reduction ≥ 20% at treatment day 14	Neuroinflammation	Its plasma level shows diagnostic value for MDD (AUC = 0.796, Se = 60.4%, Sp = 97.9%); patients with ≥ 20% decline on day14 achieve 78.3% 8-week remission, while those with < 20% decline only reach 32.6% remission	[117]

(↑=increase; ↓=decrease)

### Epigenetic-targeted therapeutic strategies for depression

For DNA methylation-targeted therapeutic interventions in MDD, evidence from both preclinical and clinical studies has linked dynamic DNA methylation dysregulation to behavioural phenotypes associated with depression. In the learned helplessness (LH) mouse model, chronic stress induces DNA hypermethylation in the dorsal hippocampus and prefrontal cortex (PFC), alongside upregulated expression of *DNMT3a* and *DNMT3b*. Although imipramine alleviates escape deficits in mice under both acute and chronic dosing regimens, only chronic imipramine administration specifically reverses stress-triggered DNA methylation aberrations and abnormal DNMT expression within the PFC [118]. Clinically, a small human trial of a small human trial of the traditional Chinese medicine Xiaoyao Wan demonstrated that it effectively relieves psychomotor retardation in patients with mild-to-moderate MDD, an effect associated with upregulated *DNMT1* expression and reversal of genome-wide aberrant DNA methylation [119]. Collectively, these findings suggest that *DNMT* subtype‑specific modulators could represent a future direction for precision MDD therapy; however, such approaches remain at an experimental stage and require further preclinical optimisation and clinical validation.

HDAC inhibitors and advanced delivery systems, Preclinical studies have demonstrated that systemic or region‑specific administration of histone deacetylase inhibitors ameliorates depressive‑like behaviours in animal models. Specifically, local injection of the HDAC inhibitor MS-275 into the NAc increases histone H3/H4 acetylation and restores physiological function of the mesolimbic reward circuit [66]. Subtype-specific mechanistic studies further indicate that inhibition of Class I HDACs (*HDAC1/HDAC3*) primarily mediates antidepressant effects, whereas concurrent inhibition of Class II HDACs (*HDAC5*) augments anxiolytic properties in rodent paradigms. Moreover, combination therapy with fluoxetine and HDACis synergistically enhances histone H4 acetylation at the *BDNF* promoter, promotes RNA polymerase II recruitment, and thus markedly boosts *BDNF* transcriptional activity [120]. In recent years, novel subtype‑selective HDAC inhibitors have been developed in preclinical settings. T2943, a 2024-reported HDAC5-selective inhibitor, crosses the blood-brain barrier (BBB), upregulates hippocampal H3K14ac levels, reverses depressive-like phenotypes, and exhibits no hematological toxicity in mouse models [121]. In a 2025 proof‑of‑concept study, an extracellular vesicle(EV)‑mediated delivery system was engineered with transferrin receptor‑targeting peptides to improve BBB penetration and enable precise microglial targeting. In a lipopolysaccharide(LPS)‑induced mouse model of depressive‑like behaviours, this system co‑delivered *HDAC5*‑targeted siRNA and metal‑based nanozymes, resulting in mitigated neuroinflammation and depressive‑like phenotypes [122]. While this system offers a promising preclinical strategy for CNS‑targeted epigenetic therapy, its translational potential remains to be systematically evaluated in clinical settings.

For targeted regulation of histone methylation, Preclinical evidence indicates that early-life stress in mice upregulates *DOT1L* expression in the NAc, increases H3K79me2 enrichment, and represses the transcription of neurotrophic genes such as *BDNF*. Pharmacological inhibition of *DOT1L* via EPZ-5676 reduces H3K79me2 enrichment and ameliorates social avoidance and anhedonic behavioral phenotypes in mice [123]. In a preclinical mouse model of chronic social defeat stress, sserotonin within the dorsal raphe nucleus (DRN) directly mediates the histone H3K4me3Q5ser modification, regulating the transcription of stress-related genes *CRHR1* and *FKBP5*. Chronic stress markedly downregulates this histone modification, leading to dysregulated transcription of stress-related genes and subsequent development of depressive-like phenotypes. Nevertheless, SSRI-based pharmacotherapy or CRISPR-mediated gene editing to restore this epigenetic modification rescues chromatin accessibility, normalizes stress-related gene transcription, and thereby reverses depressive-like phenotypes in rodent models [124]. While these findings uncover a novel epigenetic role for serotonin and provide a theoretical foundation for neurotransmitter‑epigenetic dual‑target strategies, both *DOT1L* inhibition and CRISPR‑based approaches are at an early preclinical stage, and their safety and efficacy in human MDD have yet to be established.

Early‑stage clinical studies have identified non‑coding RNA‑based strategies as a potential avenue for MDD treatment. In a single-center exploratory RCT of 78 untreated MDD patients, baseline plasma miR-483-5p levels showed the most robust inverse correlation with HAM-D improvement at week 2 of SSRI treatment. In silico pathway analyses further predicted interactions between this miRNA and the TGF-β signaling pathway, including SMAD-related genes. These preliminary findings suggest miR-483-5p as a candidate predictive marker for early SSRI response and point to a potential mechanistic involvement, warranting independent validation in larger cohorts before clinical translation [73]. In a clinical study of MDD patients, 141 miRNAs were significantly downregulated in plasma after 4 weeks of mirtazapine monotherapy; among these, miR-1237-5p modulates therapeutic response by targeting the MAPK/IGF signaling pathway, and its expression level is negatively correlated with clinical remission [125]. In a clinical study of untreated MDD patients, lower pre‑treatment miR‑4707‑3p expression was linked to SSRI remission, whereas higher miR‑6068 expression was associated with mirtazapine remission and greater symptom improvemen [126]. These findings highlight miRNA‑driven mitochondrial regulatory mechanisms as a promising research direction for understanding antidepressant response, although further validation in larger cohorts is required.

Non-pharmacological interventions such as physical exercise exert antidepressant effects by remodeling epigenetic signatures across key brain regions. Preclinical studies have demonstrated that 10-day treadmill training reverses stress-induced aberrations in histone H3 acetylation within the anterior cingulate cortex (ACC) and insular cortex (IC), while downregulating the expression of *HDAC1*, FosB, and p-CREB, thereby ameliorating anxiety-and pain-associated depressive-like behaviors in mice. However, although short-term exercise training may alleviate anxiety-like symptoms, incomplete epigenetic remodeling within the insula may exacerbate pain-related behavioral phenotypes [127]. These findings suggest that exercise‑based interventions may require sufficient duration to achieve coordinated epigenetic remodelling, offering preclinical insights for future non‑pharmacological strategies. In high-fat diet-induced obese postpartum mouse models, inulin‑rich dietary fibre boosted gut short‑chain fatty acids (SCFAs) as endogenous HDAC inhibitors, driving epigenetic restoration of brain plasticity and relieving depressive‑like behaviours [128]. In a clinical context, Silva et al. illustrated that trauma-focused psychotherapies, particularly EMDR, could remodel peripheral DNA methylation landscapes in treatment-resistant depression patients with early-life adversity; such epigenetic rewiring primarily targets inflammatory genes including LTA and S100A8, and rectifies aberrant methylation within TNF signaling cascades to erase depression-associated inflammatory epigenetic signaturesv [129].

Collectively, various epigenetic modulators and non-drug interventions act on different epigenetic regulatory layers to reverse stress-induced molecular epigenetic disorders and rescue depressive pathological phenotypes (Table 4.). Most related intervention schemes are still restricted to preclinical animal research, while only a few therapeutic approaches have obtained preliminary human trial evidence. Further large-scale clinical verification and optimization of targeted delivery tools are essential to advance the clinical transformation of epigenetic antidepressant strategies.

**Table 4:** Summary of epigenetic therapeutic targets and corresponding interventions for MDD Species: Human, rodent

Epigenetic Target	Biospecimen	Representative Drugs/ Interventions	Core Molecular Mechanism	Antidepressant Effects	References
DNMT	Prefrontal cortex, hippocampus; human peripheral blood	Chronic imipramine, Xiaoyao Wan	Abnormal DNA methylation←reversed→DNMT subtype balance restored	PFC hypermethylation ↓→stress escape deficits↓;global methylation disorder ↓→psychomotor retardation↓	[118, 119]
HDAC	Nucleus accumbens, hippocampus; microglia; human peripheral blood	MS-275,T2943, fluoxetine combined HDAC inhibitors, EV-HDAC5 siRNA nanozyme	Histone acetylation ↑ → BDNF transcription ↑; EV carrier → BBB penetration & microglia targeting	Reward circuit function↑→depressive behaviors↓; neuroinflammation↓ →LPS-induced depression↓	[66, 120–122]
Histone methylation	Nucleus accumbens, dorsal raphe; post-mortem human brain	EPZ-5676,SSRIs, CRISPR editing	H3K79me2↓/ H3K4me3Q5ser restoration→chromatin accessibility↑	Neurotrophic gene transcription↑→anhedonia&social avoidance↓;stress gene disorder↓	[123, 124]
ncRNA	Human plasma, peripheral blood; rodent brain	miR-483-5p, mirtazapine, SSRI regimens	miRNA modulation → TGF-β / MAPK / mitochondrial pathway remodeling	Clinical remission positively correlated with altered miRNA levels	[73, 125, 126]
Non-pharmacological intervention	Anterior cingulate, insula; human peripheral blood	Treadmill exercise, inulin-rich diet, EMDR psychotherapy	Histone acetylation normalized / gut SCFAs ↑/inflammatory methylation reprogramming	Anxiety& pain-related depressive symptoms ↓; brain plasticity ↑;inflammatory epigenetic signatures erased	[127–129]

(↑=upregulate; ↓=downregulate; →=lead to)

### Current limitations and translational bottlenecks

Current epigenetic studies have clarified the pathogenic roles of core regulatory mechanisms including DNA methylation and histone modifications in MDD. Nevertheless, translating these research findings into clinical diagnosis and treatment still faces notable challenges. The primary barrier lies in the spatial heterogeneity of epigenetic marks, which manifests at two levels: tissue type and cell type. Both forms of heterogeneity greatly restrict clinical translation.

On the one hand, brain regions such as the hippocampus and prefrontal cortex represent the primary pathological lesions in MDD and serve as key targets for epigenetic-oriented clinical diagnosis and intervention. Due to ethical constraints and limited clinical access to human brain tissue, most existing clinical studies rely on surrogate specimens including peripheral blood and saliva to indirectly reflect brain-specific epigenetic changes. Nevertheless, methylation patterns in peripheral tissues are poorly consistent with those in brain tissue and may even show opposite regulatory trends. Such inconsistency directly reduces the reliability of peripheral surrogate samples for guiding clinical diagnosis and intervention. For instance, the *Bicd2* gene exhibits hypermethylation in peripheral blood but hypomethylation in the hippocampus of CUMS mice [30]. Integrating multi-omic datasets across 54 eligible transcriptomic and methylomic studies (30 brain-only, 20 blood-only, and 4 dual brain-blood cohorts), Zallar and Dupont identified 744 concordantly dysregulated differentially expressed genes (DEGs) and 544 consistently altered differentially methylated genes (DMGs shared between brain and peripheral blood). Strikingly, only 43 of these shared DEGs and 34 of the overlapping DMGs coincided with established MDD risk loci derived from genome-wide association studies (GWAS) [130]. Systematic efforts to quantify blood–brain epigenetic relationships have further underscored the limitations of cross-tissue concordance. Edgar et al. developed BECon, a web-based tool that systematically evaluated DNA methylation concordance between paired human blood and three brain regions using 450 K array data. Their analysis revealed that tissue differences are one of the largest contributors to variability in the human DNA methylome, and that the connection between blood–brain DNA methylation is tenuous and not well-documented [131]. Such limited molecular congruence between central and peripheral compartments, coupled with substantial tissue- and study-specific molecular heterogeneity, strongly suggests that currently available blood molecular signatures lack sufficient robustness to act as clinically actionable surrogate biomarkers. Although cross-tissue epigenetic analysis can identify CpG sites conserved between central and peripheral tissues [132], peripheral samples still fail to capture brain-specific epigenetic variations [133]. Furthermore, different peripheral tissues vary significantly in their suitability as surrogate biospecimens for MDD epigenetic research, which further complicates the selection of clinical surrogate samples. For instance, the association between *NR3C1* methylation and MDD in mother-infant cohorts can be reliably detected in peripheral blood [23, 32], but cannot be replicated in umbilical cord blood [134]. This indicates that in clinical epigenetic research, the selection of surrogate tissues must align with the functional characteristics of the target gene [135], which is a key prerequisite for ensuring the clinical translatability of research findings. On the other hand, brain tissue shows strong cellular heterogeneity. Distinct cell types such as neurons and microglia possess highly specialized epigenetic methylation patterns, and such cell-type-specific differences further restrict the clinical value of epigenetic data obtained from bulk sequencing. Dysregulation of these cell-specific methylation signatures concentrates on key signaling pathways related to neurotrophic support and immune-mediated inflammation [136]. Conventional bulk sequencing fully masks these cell-specific epigenetic signals [137], making it difficult to identify reliable epigenetic targets for clinical intervention. Even in peripheral samples, variations in cellular composition can produce thousands of methylation changes [138]. Bulk sequencing also fails to detect region‑specific shifts in the expression of epigenetic enzymes [139]. Both issues reduce the reliability of epigenetic data for guiding clinical practice. Single-cell sequencing has uncovered epigenetic alterations specific to distinct cell subpopulations [140]. These findings further illustrate the inherent limitations of traditional bulk sequencing in clinical epigenetic research and directly support the need to adopt single-cell technologies to advance clinical translation.

Beyond the key limitations caused by spatial heterogeneity described above, current epigenetic studies on MDD encounter several prominent challenges in causal inference, dynamic trajectory characterization, result reproducibility, and clinical translation. These obstacles collectively impede the transition from basic research findings to real-world clinical applications. First, most existing studies only conduct correlational analyses of epigenetic abnormalities and depressive phenotypes, while the causal relationship between them remains unclear. Such uncertainty directly slows the development and clinical translation of targeted epigenetic therapeutic strategies. Although *BDNF* promoter hypermethylation is significantly associated with suicide risk among MDD patients [16], it is still unclear whether these methylation changes act as an upstream driver of disease susceptibility or a secondary outcome of pathological processes such as chronic stress. Recently, a large-scale methylome-wide association study (MWAS) published in Nature Mental Health applied Mendelian randomisation (MR) to infer causality. It identified 23 CpG sites with potential causal links to MDD, of which 7 were replicated in independent datasets, with the direction consistently indicating that DNA methylation drives MDD risk. The replicated signals were predominantly enriched in the MHC region (DDR1) and the GPR133 locus. Although this study offers a paradigm for causal inference, its modest effect sizes, reliance on peripheral blood, and between‑cohort heterogeneity suggest that clinical translation remains premature [42].

Major gaps persist in demographic-stratified epigenetic investigations of MDD, given marked ancestry-related heterogeneity across epigenetic molecular profiles. A systematic review and meta-analysis by Zhu et al., incorporating 47 eligible studies and 3 pooled datasets, reported that BDNF hypermethylation correlated with elevated depression risk exclusively among Asian individuals, without comparable associations detected in other ancestral groups [17]. A large-scale methylome-wide association study from Shen et al. further illustrated such population discrepancies, enrolling 24,754 European participants alongside an East Asian subcohort (243 cases, 1,846 controls). Though weak positive correlation (*r* = 0.48) was found for significant MDD-associated CpG effect sizes between two ancestral groups, no methylome-wide significant signals were uncovered in the East Asian subset [42]. Collectively, these datasets suggest population-specific epigenetic patterns linked to MDD. Even so, the field lacks adequately powered longitudinal cohorts stratified by sex and ancestry to evaluate the cross-population generalizability of such epigenetic findings. Furthermore, most existing evidence originates from small cross-sectional studies, and large longitudinal datasets tracking epigenetic fluctuations throughout MDD courses by demographic subgroups are scarce [141].

Second, epigenetic modifications are highly dynamic and reversible, and their regulatory patterns are strongly dependent on specific time windows. Such temporal dependence is a critical factor to consider when translating epigenetic interventions into clinical practice. However, inherent drawbacks of current clinical study designs prevent comprehensive profiling of these dynamic epigenetic signatures, which further reduces the precision of epigenetic targets for clinical intervention. Most existing clinical studies use cross-sectional designs. These static observational methods cannot fully depict epigenetic regulatory trajectories across the whole course of MDD, and therefore cannot provide reliable time-window guidance for clinical epigenetic interventions. Studies have demonstrated that early-life adverse stress can induce abnormal methylation at specific CpG sites within the *NR3C1* promoter in offspring and disrupt the HPA-axis stress response system, whereas positive maternal care can mitigate these adverse outcomes [142]. Nevertheless, large-scale longitudinal clinical cohorts are still needed to systematically verify the long-term dynamic epigenetic changes involved in this process. Meanwhile, epigenetic reversal triggered by treatment also shows obvious time-dependent features. Chronic imipramine treatment can specifically reverse stress-induced hypermethylation in the prefrontal cortex [118], and exercise-based interventions take more than 10 days to produce stable antidepressant effects through histone acetylation modulation [127]. These findings underline the importance of optimizing intervention timing for clinical epigenetic therapies. Neglecting such time-dependent characteristics in clinical study designs may lead to one-sided interpretations and misjudgments of epigenetic intervention efficacy, which directly weakens the clinical translatability of research outcomes.

Considerable heterogeneity in clinical research protocols directly results in low reproducibility of relevant findings and further compromises their clinical translational potential. Whole-genome bisulfite sequencing (WGBS) serves as a representative example. Its analytical outcomes are directly affected by library construction procedures, sequencing platforms, and bioinformatic pipelines. Compared with methylation array data, WGBS contains inherent systematic bias and requires ultra-high sequencing coverage (> 100×) to obtain robust single-base-resolution results [143]. Its high-cost nature also severely limits large-scale applications in large-cohort clinical trials and observational studies. In addition, inconsistent protocols for RNA extraction, primer design, and quantitative analysis in non-coding RNA (ncRNA) detection cause substantial inter-study variation in results, which greatly reduces the comparability of ncRNA-related epigenetic evidence across different investigations. Finally, despite the identification of multiple epigenetic biomarkers and therapeutic targets with promising clinical translational potential, their implementation in routine clinical practice still faces several major obstacles. Most candidate epigenetic biomarkers have only been validated in single-center, small-sample retrospective studies, without systematic verification through multicenter, large-cohort prospective clinical trials. Their diagnostic sensitivity, specificity and predictive performance have not been validated across diverse age groups, genders, ethnic populations and MDD subtypes. Moreover, unified industry-wide standards have not yet been established regarding the methodological framework, reference ranges and interpretation criteria for epigenetic diagnostic assays. Considerable differences in testing workflows and quality-control systems among clinical laboratories have become a key bottleneck preventing these biomarkers from being incorporated into routine clinical diagnostic workflows.

## Summary and future translational outlook

Epigenetic studies on MDD have identified five core regulatory mechanisms that govern disease onset and progression. These mechanisms include DNA methylation, histone modifications, ncRNA-mediated regulation, RNA chemical modifications, and chromatin remodeling. In DNA methylation studies, aberrant methylation of core regulatory genes such as *BDNF*, *NR3C1*, and *SLC6A4* has been linked to MDD progression, with potential involvement of pathways regulating neuronal plasticity, HPA axis homeostasis, and neurotransmitter transport. These epigenetic changes exhibit strict tissue- and cell-type specificity and are influenced by critical developmental windows, especially early-life adversity. The identification of additional regulatory genes such as *KLK8* and *FKBP5* has further improved the DNA methylation-driven regulatory network underlying MDD. In terms of histone modifications, acetylation imbalances mediated by the HDAC family and abnormal histone methylation (e.g., H3K27me3/H3K4me3) regulate the transcription of key genes such as BDNF by reshaping chromatin structure. Emerging histone marks, including crotonylation, also provide new approaches to decipher MDD-associated pathogenic mechanisms. NcRNAs regulate MDD-related neurogenesis and neuroinflammatory signaling through post-transcriptional mechanisms, and they exhibit distinct brain-region- and cell-type-specific expression patterns. RNA chemical modifications (e.g., m⁶A, ac4C, m⁵C) modulate neuronal function by altering RNA metabolism, forming a complex epigenetic transcriptional regulatory network in MDD pathogenesis. Chromatin-remodeling complexes control the transcription of genes related to neuroplasticity and energy metabolism by regulating nucleosome positioning. Antidepressants such as fluoxetine exert therapeutic effects by normalizing epigenetic abnormalities associated with chromatin remodeling.

From a clinical translational perspective, epigenetic biomarkers hold considerable potential for MDD diagnosis and early prediction of therapeutic response. Epigenetic-targeted therapeutic interventions include HDAC subtype-selective inhibitors, RNA-targeted modulators, and EV-based CNS-targeted delivery systems, which provide novel strategies to improve therapeutic efficacy in MDD. Nevertheless, current translational research confronts four core bottlenecks. These bottlenecks include the tissue-and cell-type heterogeneity of epigenetic signatures between peripheral blood and central nervous system tissues, unclear causal relationships between epigenetic dysregulation and depressive-like behavioral phenotypes, low methodological reproducibility, and the absence of unified clinical-translational standardization frameworks.

Future research should employ single-cell and spatial multi-omics approaches to decipher cell-type-specific epigenetic regulatory mechanisms and identify blood-detectable, tissue-conserved epigenetic diagnostic biomarkers. Large-scale multicenter prospective clinical validation and the development of individualized epigenetic-targeted therapeutic regimens will accelerate the translational process of MDD epigenetics, facilitating its advancement from basic mechanistic discoveries to clinical precision diagnosis and personalized therapy.

## Data Availability

All data included in this review are derived from publicly available published articles cited in the manuscript.

## References

[1] Ferrari AJ, Santomauro DF, Aali A, Abate YH. Global incidence, prevalence, years lived with disability (YLDs), disability-adjusted life-years (DALYs), and healthy life expectancy (HALE) for 371 diseases and injuries in 204 countries and territories and 811 subnational locations, 1990–2021: a systematic analysis for the Global Burden of Disease Study 2021. Lancet. 2024;403:2133–61. 10.1016/S0140-6736(24)00757-8. 38642570 10.1016/S0140-6736(24)00757-8 PMC11122111

[2] Marx W, Penninx BWJH, Solmi M, Furukawa TA, Firth J, Carvalho AF, Berk M. Major depressive disorder. Nat Rev Dis Primers. 2023;9:44. 10.1038/s41572-023-00454-1. 37620370 10.1038/s41572-023-00454-1

[3] Šalamon Arčan I, Kouter K, Videtič A, Paska. Depressive disorder and antidepressants from an epigenetic point of view. World J Psychiatry. 2022;12:1150–68. 10.5498/wjp.v12.i9.1150. 36186508 10.5498/wjp.v12.i9.1150 PMC9521527

[4] Berger SL, Kouzarides T, Shiekhattar R, Shilatifard A. An operational definition of epigenetics. Genes Dev. 2009;23:781–3. 10.1101/gad.1787609. 19339683 10.1101/gad.1787609 PMC3959995

[5] Sullivan PF, Neale MC, Kendler KS. Genetic epidemiology of major depression: review and meta-analysis. Am J Psychiatry. 2000;157:1552–62. 10.1176/appi.ajp.157.10.1552. 11007705 10.1176/appi.ajp.157.10.1552

[6] Fraga MF, Ballestar E, Paz MF, Ropero S, Setien F, Ballestar ML, Heine-Suñer D, Cigudosa JC, Urioste M, Benitez J, Boix-Chornet M, Sanchez-Aguilera A, Ling C, Carlsson E, Poulsen P, Vaag A, Stephan Z, Spector TD, Wu Y-Z, Plass C, Esteller M. Epigenetic differences arise during the lifetime of monozygotic twins. Proc Natl Acad Sci U S A. 2005;102:10604–9. 10.1073/pnas.0500398102. 16009939 10.1073/pnas.0500398102 PMC1174919

[7] Alshaya DS. Genetic and epigenetic factors associated with depression: An updated overview. Saudi J Biol Sci. 2022;29:103311. 10.1016/j.sjbs.2022.103311. 35762011 10.1016/j.sjbs.2022.103311 PMC9232544

[8] Dudley KJ, Li X, Kobor MS, Kippin TE, Bredy TW. Epigenetic mechanisms mediating vulnerability and resilience to psychiatric disorders. Neurosci Biobehav Rev. 2011;35:1544–51. 10.1016/j.neubiorev.2010.12.016. 21251925 10.1016/j.neubiorev.2010.12.016

[9] Aljabali AAA, Alkaraki AK, Gammoh O, Tambuwala MM, Mishra V, Mishra Y, Hassan SS. El-Tanani, Deciphering Depression: Epigenetic Mechanisms and Treatment Strategies. Biology (Basel). 2024;13:638. 10.3390/biology13080638. 39194576 10.3390/biology13080638 PMC11351889

[10] Jones PA. Functions of DNA methylation: islands, start sites, gene bodies and beyond. Nat Rev Genet. 2012;13:484–92. 10.1038/nrg3230. 22641018 10.1038/nrg3230

[11] Wu SC, Zhang Y, Active DNA. demethylation: many roads lead to Rome. Nat Rev Mol Cell Biol. 2010;11:607–20. 10.1038/nrm2950. 20683471 10.1038/nrm2950 PMC3711520

[12] He Y-F, Li B-Z, Li Z, Liu P, Wang Y, Tang Q, Ding J, Jia Y, Chen Z, Li L, Sun Y, Li X, Dai Q, Song C-X, Zhang K, He C, Xu G-L. Tet-mediated formation of 5-carboxylcytosine and its excision by TDG in mammalian DNA. Science. 2011;333:1303–7. 10.1126/science.1210944. 21817016 10.1126/science.1210944 PMC3462231

[13] Guo JU, Su Y, Shin JH, Shin J, Li H, Xie B, Zhong C, Hu S, Le T, Fan G, Zhu H, Chang Q, Gao Y, Ming G, Song H. Distribution, recognition and regulation of non-CpG methylation in the adult mammalian brain. Nat Neurosci. 2014;17:215–22. 10.1038/nn.3607. 24362762 10.1038/nn.3607 PMC3970219

[14] Greenberg MVC, Bourc’his D. The diverse roles of DNA methylation in mammalian development and disease. Nat Rev Mol Cell Biol. 2019;20:590–607. 10.1038/s41580-019-0159-6. 31399642 10.1038/s41580-019-0159-6

[15] Ferrer A, Labad J, Salvat-Pujol N, Barrachina M, Costas J, Urretavizcaya M, de Arriba-Arnau A, Crespo JM, Soriano-Mas C, Carracedo Á, Menchón JM, Soria V. BDNF genetic variants and methylation: effects on cognition in major depressive disorder. Transl Psychiatry. 2019;9:265. 10.1038/s41398-019-0601-8. 31636250 10.1038/s41398-019-0601-8 PMC6803763

[16] Kang H-J, Kim J-M, Lee J-Y, Kim S-Y, Bae K-Y, Kim S-W, Shin I-S, Kim H-R, Shin M-G, Yoon J-S. BDNF promoter methylation and suicidal behavior in depressive patients. J Affect Disord. 2013;151:679–85. 10.1016/j.jad.2013.08.001. 23992681 10.1016/j.jad.2013.08.001

[17] Zhu J-H, Bo H-H, Liu B-P, Jia C-X. The associations between DNA methylation and depression: A systematic review and meta-analysis. J Affect Disord. 2023;327:439–50. 10.1016/j.jad.2023.01.079. 36717033 10.1016/j.jad.2023.01.079

[18] Song Y, Miyaki K, Suzuki T, Sasaki Y, Tsutsumi A, Kawakami N, Shimazu A, Takahashi M, Inoue A, Kan C, Kurioka S, Shimbo T. Altered DNA methylation status of human brain derived neurotrophis factor gene could be useful as biomarker of depression. Am J Med Genet B Neuropsychiatr Genet. 2014;165B:357–64. 10.1002/ajmg.b.32238. 24801253 10.1002/ajmg.b.32238 PMC4321058

[19] Boersma GJ, Lee RS, Cordner ZA, Ewald ER, Purcell RH, Moghadam AA, Tamashiro KL. Prenatal stress decreases Bdnf expression and increases methylation of Bdnf exon IV in rats. Epigenetics. 2014;9:437–47. 10.4161/epi.27558. 24365909 10.4161/epi.27558 PMC4053462

[20] Cheng S, Wang W, Zhu Z, Zhao M, Li H, Liu D, Pan F. Involvement of brain-derived neurotrophic factor methylation in the prefrontal cortex and hippocampus induced by chronic unpredictable mild stress in male mice. J Neurochem. 2023;164:624–42. 10.1111/jnc.15735. 36453259 10.1111/jnc.15735

[21] Niknazar S, Nahavandi A, Peyvandi AA, Peyvandi H, Akhtari AS, Karimi M. Comparison of the Adulthood Chronic Stress Effect on Hippocampal BDNF Signaling in Male and Female Rats. Mol Neurobiol. 2016;53:4026–33. 10.1007/s12035-015-9345-5. 26189832 10.1007/s12035-015-9345-5

[22] McGowan PO, Sasaki A, D’Alessio AC, Dymov S, Labonté B, Szyf M, Turecki G, Meaney MJ. Epigenetic regulation of the glucocorticoid receptor in human brain associates with childhood abuse. Nat Neurosci. 2009;12:342–8. 10.1038/nn.2270. 19234457 10.1038/nn.2270 PMC2944040

[23] Wei L, Ying X, Zhai M, Li J, Liu D, Liu X, Yu B, Yan H. The association between peritraumatic distress, perceived stress, depression in pregnancy, and NR3C1 DNA methylation among Chinese pregnant women who experienced COVID-19 lockdown. Front Immunol. 2022;13:966522. 10.3389/fimmu.2022.966522. 36091061 10.3389/fimmu.2022.966522 PMC9453447

[24] Bakusic J, Vrieze E, Ghosh M, Bekaert B, Claes S, Godderis L. Increased methylation of NR3C1 and SLC6A4 is associated with blunted cortisol reactivity to stress in major depression. Neurobiol Stress. 2020;13:100272. 10.1016/j.ynstr.2020.100272. 33344725 10.1016/j.ynstr.2020.100272 PMC7739183

[25] Olsson CA, Foley DL, Parkinson-Bates M, Byrnes G, McKenzie M, Patton GC, Morley R, Anney RJL, Craig JM, Saffery R. Prospects for epigenetic research within cohort studies of psychological disorder: a pilot investigation of a peripheral cell marker of epigenetic risk for depression. Biol Psychol. 2010;83:159–65. 10.1016/j.biopsycho.2009.12.003. 20018225 10.1016/j.biopsycho.2009.12.003

[26] Philibert R, Madan A, Andersen A, Cadoret R, Packer H, Sandhu H. Serotonin transporter mRNA levels are associated with the methylation of an upstream CpG island. Am J Med Genet B Neuropsychiatr Genet. 2007;144B:101–5. 10.1002/ajmg.b.30414. 16958039 10.1002/ajmg.b.30414

[27] Sanwald S, Widenhorn-Müller K, Schönfeldt-Lecuona C, Montag C, Kiefer M. Factors related to age at depression onset: the role of SLC6A4 methylation, sex, exposure to stressful life events and personality in a sample of inpatients suffering from major depression. BMC Psychiatry. 2021;21:167. 10.1186/s12888-021-03166-6. 33765975 10.1186/s12888-021-03166-6 PMC7995700

[28] Kao W-T, Chang C-L, Lung F-W. 5-HTT mRNA level as a potential biomarker of treatment response in patients with major depression in a clinical trial. J Affect Disord. 2018;238:597–608. 10.1016/j.jad.2018.06.035. 29957477 10.1016/j.jad.2018.06.035

[29] Oe S, Miki H, Nishimura W, Noda Y. Mechanism of the Dendritic Translation and Localization of Brain-derived Neurotrophic Factor. Cell Struct Funct. 2016;41:23–31. 10.1247/csf.15015. 26700412 10.1247/csf.15015

[30] Xiu J, Li J, Liu Z, Wei H, Zhu C, Han R, Liu Z, Zhu W, Shen Y, Xu Q. Elevated BICD2 DNA methylation in blood of major depressive disorder patients and reduction of depressive-like behaviors in hippocampal Bicd2-knockdown mice. Proc Natl Acad Sci U S A. 2022;119:e2201967119. 10.1073/pnas.2201967119. 35858435 10.1073/pnas.2201967119 PMC9335189

[31] Starnawska A, Bukowski L, Chernomorchenko A, Elfving B, Müller HK, van den Oord E, Aberg K, Guintivano J, Grove J, Mors O, Børglum AD, Nielsen AL, Qvist P, Staunstrup NH. DNA methylation of the KLK8 gene in depression symptomatology. Clin Epigenetics. 2021;13:200. 10.1186/s13148-021-01184-5. 34715912 10.1186/s13148-021-01184-5 PMC8556955

[32] Mendonça MS, Mangiavacchi PM, Mendes AV, Loureiro SR, Martín-Santos R, Glória LS, Marques W, De Marco SPG, Kanashiro MM, Hallak JEC, Crippa JAS, Rios ÁFL. DNA methylation in regulatory elements of the FKBP5 and NR3C1 gene in mother-child binomials with depression. J Affect Disord. 2023;331:287–99. 10.1016/j.jad.2023.03.031. 36933666 10.1016/j.jad.2023.03.031

[33] Na K-S, Won E, Kang J, Kim A, Choi S, Tae W-S, Kim Y-K, Lee M-S, Joe S-H, Ham B-J. Differential effect of COMT gene methylation on the prefrontal connectivity in subjects with depression versus healthy subjects. Neuropharmacology. 2018;137:59–70. 10.1016/j.neuropharm.2018.04.030. 29723539 10.1016/j.neuropharm.2018.04.030

[34] Tesfaye M, Chatterjee S, Zeng X, Joseph P, Tekola-Ayele F. Impact of depression and stress on placental DNA methylation in ethnically diverse pregnant women. Epigenomics. 2021;13:1485–96. 10.2217/epi-2021-0192. 34585950 10.2217/epi-2021-0192 PMC8503803

[35] Hing B, Sathyaputri L, Potash JB. A comprehensive review of genetic and epigenetic mechanisms that regulate BDNF expression and function with relevance to major depressive disorder. Am J Med Genet B Neuropsychiatr Genet. 2018;177:143–67. 10.1002/ajmg.b.32616. 29243873 10.1002/ajmg.b.32616

[36] Oberlander TF, Weinberg J, Papsdorf M, Grunau R, Misri S, Devlin AM. Prenatal exposure to maternal depression, neonatal methylation of human glucocorticoid receptor gene (NR3C1) and infant cortisol stress responses. Epigenetics. 2008;3:97–106. 10.4161/epi.3.2.6034. 18536531 10.4161/epi.3.2.6034

[37] López-Echeverri YP, Cardona-Londoño KJ, Garcia-Aguirre JF, Orrego-Cardozo M. Effects of serotonin transporter and receptor polymorphisms on depression. Rev Colomb Psiquiatr (Engl Ed). 2021:S0034–7450(21)00135-9. 10.1016/j.rcp.2021.07.006. 10.1016/j.rcpeng.2021.07.003 37453823

[38] Goldenthal AR, Lieberman E, Rizk MM, Ogden RT, Rubin-Falcone H, Zanderigo F, Huang Y-Y, Min E, Yuan M, Milak M, Sullivan GM, Sublette ME, Oquendo MA, Mann JJ, Miller JM. Relationships between serotonin 1A receptor DNA methylation, self-reported history of childhood abuse and gray matter volume in major depression. J Affect Disord. 2024;367:307–17. 10.1016/j.jad.2024.08.148. 39187183 10.1016/j.jad.2024.08.148 PMC11558534

[39] Delahaye F, Do C, Kong Y, Ashkar R, Salas M, Tycko B, Wapner R, Hughes F. Genetic variants influence on the placenta regulatory landscape. PLoS Genet. 2018;14:e1007785. 10.1371/journal.pgen.1007785. 30452450 10.1371/journal.pgen.1007785 PMC6277118

[40] Yang H-H, Han K-M, Kim A, Kang Y, Tae W-S, Han M-R, Ham B-J. Neuroimaging and epigenetic analysis reveal novel epigenetic loci in major depressive disorder. Psychol Med. 2024;54:2585–98. 10.1017/S0033291724000709. 38721773 10.1017/S0033291724000709

[41] Stavrum A-K, Sirignano L, Frid LM, Frank J, Foo JC, Spindola LM, Höffler KD, Oedegaard KJ, Haavik J, Rietschel M, Witt SH, Kessler U, Oltedal L. Le Hellard, Epigenetic and blood markers associated with response to electroconvulsive therapy in patients with depressive disorders. Transl Psychiatry. 2025;16:32. 10.1038/s41398-025-03772-y. 41339323 10.1038/s41398-025-03772-y PMC12811393

[42] Shen X, Barbu M, Caramaschi D, Arathimos R, Czamara D, David FS, Dearman A, Dilkes E, Herrera-Rivero M, Huider F, Kühn L, Lu K-C, Palviainen T, Schowe AM, Shireby G, Weihs A, Wong CCY, Davyson E, Casey H, Adams MJ, Allgaier A-K, Barber M, Burrage J, Caspi A, Costeira R, Dunn EC, Feldmann L, Frank J, Freisleder FJ, Gadd DA, Greimel E, Hannon E, Harris SE, Homuth G, Howard DM, Iurato S, Korhonen T, Lu T-P, Martin NG, Martins J, McDermott E, Meinert S, Navarro P, Ollikainen M, Pehl V, Piechaczek C, Scherff AD, Stein F, Streit F, Teumer A, Völzke H, van Dongen J, Walker RM, Yusupov N, Arseneault L, Bell JT, Berger K, Binder E, Boomsma DI, Cox SR, Dannlowski U, Evans KL, Fisher HL, Forstner AJ, Grabe HJ, Kaprio J, Kircher T, Kopf-Beck J, Kumari M, Kuo P-H, Li QS, Moffitt TE, Mulcahy H, Murphy TM, Schulte-Körne G, Mill J, Lewis CM, Wray NR, McIntosh AM. A methylome-wide association study of major depression with out-of-sample case–control classification and trans-ancestry comparison. Nat Ment Health. 2025;3:1152–67. 10.1038/s44220-025-00486-4. 41069367 10.1038/s44220-025-00486-4 PMC12504109

[43] Weaver ICG, Cervoni N, Champagne FA, D’Alessio AC, Sharma S, Seckl JR, Dymov S, Szyf M, Meaney MJ. Epigenetic programming by maternal behavior. Nat Neurosci. 2004;7:847–54. 10.1038/nn1276. 15220929 10.1038/nn1276

[44] Dunn EC, Soare TW, Zhu Y, Simpkin AJ, Suderman MJ, Klengel T, Smith ADAC, Ressler K, Relton CL. Sensitive periods for the effect of childhood adversity on DNA methylation: Results from a prospective, longitudinal study. Biol Psychiatry. 2019;85:838–49. 10.1016/j.biopsych.2018.12.023. 30905381 10.1016/j.biopsych.2018.12.023 PMC6552666

[45] Kouzarides T. Chromatin modifications and their function. Cell. 2007;128:693–705. 10.1016/j.cell.2007.02.005. 17320507 10.1016/j.cell.2007.02.005

[46] Shahbazian MD, Grunstein M. Functions of site-specific histone acetylation and deacetylation. Annu Rev Biochem. 2007;76:75–100. 10.1146/annurev.biochem.76.052705.162114. 17362198 10.1146/annurev.biochem.76.052705.162114

[47] Schwer B, Verdin E. Conserved metabolic regulatory functions of sirtuins. Cell Metab. 2008;7:104–12. 10.1016/j.cmet.2007.11.006. 18249170 10.1016/j.cmet.2007.11.006

[48] Uchida S, Hara K, Kobayashi A, Otsuki K, Yamagata H, Hobara T, Suzuki T, Miyata N, Watanabe Y. Epigenetic status of Gdnf in the ventral striatum determines susceptibility and adaptation to daily stressful events. Neuron. 2011;69:359–72. 10.1016/j.neuron.2010.12.023. 21262472 10.1016/j.neuron.2010.12.023

[49] Kenworthy CA, Sengupta A, Luz SM, Ver Hoeve ES, Meda K, Bhatnagar S, Abel T. Social defeat induces changes in histone acetylation and expression of histone modifying enzymes in the ventral hippocampus, prefrontal cortex, and dorsal raphe nucleus. Neuroscience. 2014;264:88–98. 10.1016/j.neuroscience.2013.01.024. 23370319 10.1016/j.neuroscience.2013.01.024

[50] Santos L, Possa L, Fröhlich N, Behrens L, Barbosa C, Tiefensee-Ribeiro C, Gelain DP, Almeida RF, Moreira JCF. Histone Acetylation Modifications and HDACs are Associated with Resilience or Susceptibility to Stress in a Rat Model of Major Depressive Disorder. Neurochem Res. 2025;50:196. 10.1007/s11064-025-04455-2. 40504297 10.1007/s11064-025-04455-2

[51] Fuchikami M, Morinobu S, Kurata A, Yamamoto S, Yamawaki S. Single immobilization stress differentially alters the expression profile of transcripts of the brain-derived neurotrophic factor (BDNF) gene and histone acetylation at its promoters in the rat hippocampus. Int J Neuropsychopharmacol. 2009;12:73–82. 10.1017/S1461145708008997. 18544182 10.1017/S1461145708008997

[52] Ribeiro-Davis A, Al Saeedy DY, Jahr FM, Hawkins E, McClay JL, Deshpande LS. Ketamine Produces Antidepressant Effects by Inhibiting Histone Deacetylases and Upregulating Hippocampal Brain-Derived Neurotrophic Factor Levels in a Diisopropyl Fluorophosphate-Based Rat Model of Gulf War Illness. J Pharmacol Exp Ther. 2024;388:647–54. 10.1124/jpet.123.001824. 37863487 10.1124/jpet.123.001824 PMC10801753

[53] Cortés-Erice M, Garayo-Larrea A, Fernández-Ovejero R, Aubá E, Lizaso S, Aldaz P, Bes-Rastrollo M, Gil JL, Barrado L, Ortuño F, Molero P, Tordera RM. Epigenetic, neuroplasticity, and adrenergic targets associated with major depression in immune cells. Sci Rep. 2026;16:12318. 10.1038/s41598-026-36954-9. 41786805 10.1038/s41598-026-36954-9 PMC13079730

[54] Misztak P, Pańczyszyn-Trzewik P, Nowak G, Sowa-Kućma M. Epigenetic marks and their relationship with BDNF in the brain of suicide victims. PLoS ONE. 2020;15:e0239335. 10.1371/journal.pone.0239335. 32970734 10.1371/journal.pone.0239335 PMC7513998

[55] Izzo A, Schneider R. Chatting histone modifications in mammals. Brief Funct Genomics. 2010;9:429–43. 10.1093/bfgp/elq024. 21266346 10.1093/bfgp/elq024 PMC3080777

[56] Torres-Berrío A, Estill M, Patel V, Ramakrishnan A, Kronman H, Minier-Toribio A, Issler O, Browne CJ, Parise EM, van der Zee YY, Walker DM, Martínez-Rivera FJ, Lardner CK, Durand-de R, Cuttoli SJ, Russo L, Shen S, Sidoli EJ, Nestler. Mono-methylation of lysine 27 at histone 3 confers lifelong susceptibility to stress. Neuron. 2024;112:2973–e298910. 10.1016/j.neuron.2024.06.006. 38959894 10.1016/j.neuron.2024.06.006 PMC11377169

[57] Yao Y, Du J, Wang D, Li N, Tao Z, Wu D, Peng F, Shi J, Zhou W, Zhao T, Tang Y. High-intensity interval training ameliorates postnatal immune activation-induced mood disorders through KDM6B-regulated glial activation. Brain Behav Immun. 2024;120:290–303. 10.1016/j.bbi.2024.06.006. 38851307 10.1016/j.bbi.2024.06.006

[58] Tseng C-C, Wang S-C, Yang Y-C, Fu H-C, Chou C-K, Kang H-Y, Hung Y-Y. Aberrant Histone Modification of TNFAIP3, TLR4, TNIP2, miR-146a, and miR-155 in Major Depressive Disorder. Mol Neurobiol. 2023;60:4753–60. 10.1007/s12035-023-03374-z. 37148522 10.1007/s12035-023-03374-z

[59] Cruceanu C, Alda M, Nagy C, Freemantle E, Rouleau GA, Turecki G. H3K4 tri-methylation in synapsin genes leads to different expression patterns in bipolar disorder and major depression. Int J Neuropsychopharmacol. 2013;16:289–99. 10.1017/S1461145712000363. 22571925 10.1017/S1461145712000363 PMC3564952

[60] Wu M-S, Li X-J, Liu C-Y, Xu Q, Huang J-Q, Gu S, Chen J-X. Effects of Histone Modification in Major Depressive Disorder. Curr Neuropharmacol. 2022;20:1261–77. 10.2174/1570159X19666210922150043. 34551699 10.2174/1570159X19666210922150043 PMC9881074

[61] Chwang WB, O’Riordan KJ, Levenson JM, Sweatt JD. ERK/MAPK regulates hippocampal histone phosphorylation following contextual fear conditioning. Learn Mem. 2006;13:322–8. 10.1101/lm.152906. 16741283 10.1101/lm.152906 PMC1475813

[62] Liu K, Yuan C, Li H, Chen K, Lu L, Shen C, Zheng X. A qualitative proteome-wide lysine crotonylation profiling of papaya (Carica papaya L). Sci Rep. 2018;8:8230. 10.1038/s41598-018-26676-y. 29844531 10.1038/s41598-018-26676-y PMC5974297

[63] Liu Y, Li M, Fan M, Song Y, Yu H, Zhi X, Xiao K, Lai S, Zhang J, Jin X, Shang Y, Liang J, Huang Z. Chromodomain Y-like Protein-Mediated Histone Crotonylation Regulates Stress-Induced Depressive Behaviors. Biol Psychiatry. 2019;85:635–49. 10.1016/j.biopsych.2018.11.025. 30665597 10.1016/j.biopsych.2018.11.025

[64] Wang S, Mu G, Qiu B, Wang M, Yu Z, Wang W, Wang J, Yang Y. The Function and related Diseases of Protein Crotonylation. Int J Biol Sci. 2021;17:3441–55. 10.7150/ijbs.58872. 34512158 10.7150/ijbs.58872 PMC8416722

[65] Zhang DE, Nelson DA. Histone acetylation in chicken erythrocytes. Rates of deacetylation in immature and mature red blood cells. Biochem J. 1988;250:241–5. 10.1042/bj2500241. 3355515 10.1042/bj2500241 PMC1148839

[66] Covington HE, Maze I, LaPlant QC, Vialou VF, Ohnishi YN, Berton O, Fass DM, Renthal W, Rush AJ, Wu EY, Ghose S, Krishnan V, Russo SJ, Tamminga C, Haggarty SJ, Nestler EJ. Antidepressant actions of histone deacetylase inhibitors. J Neurosci. 2009;29:11451–60. 10.1523/JNEUROSCI.1758-09.2009. 19759294 10.1523/JNEUROSCI.1758-09.2009 PMC2775805

[67] Mukhoti A, Annapoorna PK, Kumar A, Wadnerkar P, Khan AA, Pathak SS, Chakravarty S, Kumar A. Role of Repressive Histone Lysine Demethylases and Methylases in Susceptibility to Depression Using a Novel Progressive Social Defeat Stress Mouse Model. Cell Mol Neurobiol. 2025;45:78. 10.1007/s10571-025-01597-3. 40788579 10.1007/s10571-025-01597-3 PMC12339800

[68] Sun H, Kennedy PJ, Nestler EJ. Epigenetics of the Depressed Brain: Role of Histone Acetylation and Methylation. Neuropsychopharmacology. 2013;38:124–37. 10.1038/npp.2012.73. 22692567 10.1038/npp.2012.73 PMC3521990

[69] Kong J, Su X, Zhou C, Lin W, Lin A, Tang J. Micropeptides Encoded by Noncoding RNAs: Biological Functions and Roles in Diseases. Research. 2025;8:0967. 10.34133/research.0967. 41181701 10.34133/research.0967 PMC12573267

[70] Qureshi IA, Mehler MF. Emerging roles of non-coding RNAs in brain evolution, development, plasticity and disease. Nat Rev Neurosci. 2012;13:528–41. 10.1038/nrn3234. 22814587 10.1038/nrn3234 PMC3478095

[71] Gao Y-N, Zhang Y-Q, Wang H, Deng Y-L, Li N-M. A New Player in Depression: MiRNAs as Modulators of Altered Synaptic Plasticity. Int J Mol Sci. 2022;23:4555. 10.3390/ijms23094555. 35562946 10.3390/ijms23094555 PMC9101307

[72] Smalheiser NR, Lugli G, Rizavi HS, Torvik VI, Turecki G, Dwivedi Y. MicroRNA expression is down-regulated and reorganized in prefrontal cortex of depressed suicide subjects. PLoS ONE. 2012;7:e33201. 10.1371/journal.pone.0033201. 22427989 10.1371/journal.pone.0033201 PMC3302855

[73] Kato M, Ogata H, Tahara H, Shimamoto A, Takekita Y, Koshikawa Y, Nishida K, Nonen S, Higasa K, Kinoshita T. Multiple Pre-Treatment miRNAs Levels in Untreated Major Depressive Disorder Patients Predict Early Response to Antidepressants and Interact with Key Pathways. Int J Mol Sci. 2022;23:3873. 10.3390/ijms23073873. 35409234 10.3390/ijms23073873 PMC8999364

[74] Guan W, Xu D-W, Ji C-H, Wang C-N, Liu Y, Tang W-Q, Gu J-H, Chen Y-M, Huang J, Liu J-F, Jiang B. Hippocampal miR-206-3p participates in the pathogenesis of depression via regulating the expression of BDNF. Pharmacol Res. 2021;174:105932. 10.1016/j.phrs.2021.105932. 34628001 10.1016/j.phrs.2021.105932

[75] Mert A, Yucens B, Karagur ER, Akca H, Tumkaya S, Atesci FC. miRNAs in Major Depression: Possible Association of miR-17 and miR-92 with Childhood Traumas. Clin Psychopharmacol Neurosci. 2025;23:133–43. 10.9758/cpn.24.1218. 39820119 10.9758/cpn.24.1218 PMC11747731

[76] Li Y, Song W, Tong Y, Zhang X, Zhao J, Gao X, Yong J, Wang H. Isoliquiritin ameliorates depression by suppressing NLRP3-mediated pyroptosis via miRNA-27a/SYK/NF-κB axis. J Neuroinflammation. 2021;18:1. 10.1186/s12974-020-02040-8. 33402173 10.1186/s12974-020-02040-8 PMC7786465

[77] Quinn JJ, Chang HY. Unique features of long non-coding RNA biogenesis and function. Nat Rev Genet. 2016;17:47–62. 10.1038/nrg.2015.10. 26666209 10.1038/nrg.2015.10

[78] Tsagakis I, Douka K, Birds I, Aspden JL. Long non-coding RNAs in development and disease: conservation to mechanisms. J Pathol. 2020;250:480–95. 10.1002/path.5405. 32100288 10.1002/path.5405 PMC8638664

[79] Liu Z, Li X, Sun N, Xu Y, Meng Y, Yang C, Wang Y, Zhang K. Microarray profiling and co-expression network analysis of circulating lncRNAs and mRNAs associated with major depressive disorder. PLoS ONE. 2014;9:e93388. 10.1371/journal.pone.0093388. 24676134 10.1371/journal.pone.0093388 PMC3968145

[80] Cui X, Niu W, Kong L, He M, Jiang K, Chen S, Zhong A, Li W, Lu J, Zhang L. Long noncoding RNA expression in peripheral blood mononuclear cells and suicide risk in Chinese patients with major depressive disorder. Brain Behav. 2017;7:e00711. 10.1002/brb3.711. 28638716 10.1002/brb3.711 PMC5474714

[81] Issler O, van der Zee YY, Ramakrishnan A, Wang J, Tan C, Loh Y-HE, Purushothaman I, Walker DM, Lorsch ZS, Hamilton PJ, Peña CJ, Flaherty E, Hartley BJ, Torres-Berrío A, Parise EM, Kronman H, Duffy JE, Estill MS, Calipari ES, Labonté B, Neve RL, Tamminga CA, Brennand KJ, Dong Y, Shen L, Nestler EJ. Sex-Specific Role for the Long Non-coding RNA LINC00473 in Depression. Neuron. 2020;106:912–e9265. 10.1016/j.neuron.2020.03.023. 32304628 10.1016/j.neuron.2020.03.023 PMC7305959

[82] Yang Q, Li F, He AT, Yang BB. Circular RNAs: Expression, localization, and therapeutic potentials. Mol Ther. 2021;29:1683–702. 10.1016/j.ymthe.2021.01.018. 33484969 10.1016/j.ymthe.2021.01.018 PMC8116570

[83] Fischer JW, Leung AKL. CircRNAs: a regulator of cellular stress. Crit Rev Biochem Mol Biol. 2017;52:220–33. 10.1080/10409238.2016.1276882. 28095716 10.1080/10409238.2016.1276882 PMC5526226

[84] Fan C, Li Y, Lan T, Wang W, Long Y, Yu SY. Microglia secrete miR-146a-5p-containing exosomes to regulate neurogenesis in depression. Mol Ther. 2022;30:1300–14. 10.1016/j.ymthe.2021.11.006. 34768001 10.1016/j.ymthe.2021.11.006 PMC8899528

[85] Yu X, Bai Y, Han B, Ju M, Tang T, Shen L, Li M, Yang L, Zhang Z, Hu G, Chao J, Zhang Y, Yao H. Extracellular vesicle-mediated delivery of circDYM alleviates CUS-induced depressive-like behaviours. J Extracell Vesicles. 2022;11:e12185. 10.1002/jev2.12185. 35029057 10.1002/jev2.12185 PMC8758833

[86] Shi Y, Song R, Wang Z, Zhang H, Zhu J, Yue Y, Zhao Y, Zhang Z. Potential clinical value of circular RNAs as peripheral biomarkers for the diagnosis and treatment of major depressive disorder. EBioMedicine. 2021;66:103337. 10.1016/j.ebiom.2021.103337. 33862583 10.1016/j.ebiom.2021.103337 PMC8054154

[87] Jung Y, Goldman D. Role of RNA modifications in brain and behavior. Genes Brain Behav. 2018;17:e12444. 10.1111/gbb.12444. 29244246 10.1111/gbb.12444 PMC6233296

[88] Roignant J-Y, Soller M. m6A in mRNA: An Ancient Mechanism for Fine-Tuning Gene Expression. Trends Genet. 2017;33:380–90. 10.1016/j.tig.2017.04.003. 28499622 10.1016/j.tig.2017.04.003

[89] Niu J, Wang B, Wang T, Zhou T. Mechanism of METTL3-mediated m6A modification in depression-induced cognitive deficits. Am J Med Genet B Neuropsychiatr Genet. 2022;189:86–99. 10.1002/ajmg.b.32892. 35591810 10.1002/ajmg.b.32892

[90] Xu B, Li Q, Wu Y, Wang H, Xu J, Liu H, Xuan A. Mettl3-mediated m6 A modification of Lrp2 facilitates neurogenesis through Ythdc2 and elicits antidepressant-like effects. FASEB J. 2022;36:e22392. 10.1096/fj.202200133RR. 35716070 10.1096/fj.202200133RR

[91] Liu S, Xiu J, Zhu C, Meng K, Li C, Han R, Du T, Li L, Xu L, Liu R, Zhu W, Shen Y, Xu Q. Fat mass and obesity-associated protein regulates RNA methylation associated with depression-like behavior in mice. Nat Commun. 2021;12:6937. 10.1038/s41467-021-27044-7. 34836959 10.1038/s41467-021-27044-7 PMC8626436

[92] Wan X, Wang L, Khan MA, Peng L, Sun X, Yi X, Wang Z, Chen K. NAT10-mediated N4-acetylcytidine modification in KLF9 mRNA promotes adipogenesis. Cell Death Differ. 2025:1–17. 10.1038/s41418-025-01483-x. 10.1038/s41418-025-01483-x PMC12432206 40123006

[93] Guo X-F, Wang X-H, Fu Y-L, Meng Q, Huang B-Y, Yang R, Guo Y, Du Y-R, Wang X, Gao Y, Song L, Gong M, Wang S, Li Y-D, Shi H-S, Shi Y. Elevation of N-acetyltransferase 10 in hippocampal neurons mediates depression- and anxiety-like behaviors. Brain Res Bull. 2022;185:91–8. 10.1016/j.brainresbull.2022.05.004. 35550155 10.1016/j.brainresbull.2022.05.004

[94] Blaze J, Navickas A, Phillips HL, Heissel S, Plaza-Jennings A, Miglani S, Asgharian H, Foo M, Katanski CD, Watkins CP, Pennington ZT, Javidfar B, Espeso-Gil S, Rostandy B, Alwaseem H, Hahn CG, Molina H, Cai DJ, Pan T, Yao WD, Goodarzi H, Haghighi F, Akbarian S. Neuronal Nsun2 deficiency produces tRNA epitranscriptomic alterations and proteomic shifts impacting synaptic signaling and behavior. Nat Commun. 2021;12:4913. 10.1038/s41467-021-24969-x. 34389722 10.1038/s41467-021-24969-x PMC8363735

[95] Clapier CR, Iwasa J, Cairns BR, Peterson CL. Mechanisms of action and regulation of ATP-dependent chromatin-remodelling complexes. Nat Rev Mol Cell Biol. 2017;18:407–22. 10.1038/nrm.2017.26. 28512350 10.1038/nrm.2017.26 PMC8127953

[96] Ho L, Crabtree GR. Chromatin remodelling during development. Nature. 2010;463:474–84. 10.1038/nature08911. 20110991 10.1038/nature08911 PMC3060774

[97] Rayan NA, Kumar V, Aow J, Rastegar N, Lim MGL, O’Toole N, Aliwarga E, Arcego DM, Yeo HTG, Wong JY, Lee MY, Schmidt F, Haja HS, Tam WL, Zhang T-Y, Diorio J, Anacker C, Hen R, Parent C, Meaney MJ, Prabhakar S. Integrative multi-omics landscape of fluoxetine action across 27 brain regions reveals global increase in energy metabolism and region-specific chromatin remodelling. Mol Psychiatry. 2022;27:4510–25. 10.1038/s41380-022-01725-1. 36056172 10.1038/s41380-022-01725-1 PMC9734063

[98] Sun H, Damez-Werno DM, Scobie KN, Shao N-Y, Dias C, Rabkin J, Koo JW, Korb E, Bagot RC, Ahn FH, Cahill ME, Labonté B, Mouzon E, Heller EA, Cates H, Golden SA, Gleason K, Russo SJ, Andrews S, Neve R, Kennedy PJ, Maze I, Dietz DM, Allis CD, Turecki G, Varga-Weisz P, Tamminga C, Shen L, Nestler EJ. ACF chromatin-remodeling complex mediates stress-induced depressive-like behavior. Nat Med. 2015;21:1146–53. 10.1038/nm.3939. 26390241 10.1038/nm.3939 PMC4598281

[99] Zayed A, Baranowski C, Compagnion A-C, Vernochet C, Karaki S, Cuttoli RD, Saint-Jour E, Bhattacharya S, Marti F, Vanhoutte P, Yaniv M, Faure P, Barik J, Amar L, Tronche F, Parnaudeau S. SWI/SNF chromatin remodeler complex within the reward pathway is required for behavioral adaptations to stress. Nat Commun. 2022;13:1807. 10.1038/s41467-022-29380-8. 35379786 10.1038/s41467-022-29380-8 PMC8980038

[100] Wille A, Amort T, Singewald N, Sartori SB, Lusser A. Dysregulation of select ATP-dependent chromatin remodeling factors in high trait anxiety. Behav Brain Res. 2016;311:141–6. 10.1016/j.bbr.2016.05.036. 27208790 10.1016/j.bbr.2016.05.036

[101] Schröter K, Brum M, Brunkhorst-Kanaan N, Tole F, Ziegler C, Domschke K, Reif A, Kittel-Schneider S. Longitudinal multi-level biomarker analysis of BDNF in major depression and bipolar disorder. Eur Arch Psychiatry Clin Neurosci. 2020;270:169–81. 10.1007/s00406-019-01007-y. 30929061 10.1007/s00406-019-01007-y

[102] Kim J-M, Stewart R, Kang H-J, Kim S-W, Shin I-S, Kim H-R, Shin M-G, Kim J-T, Park M-S, Cho K-H, Yoon J-S. A longitudinal study of SLC6A4 DNA promoter methylation and poststroke depression. J Psychiatr Res. 2013;47:1222–7. 10.1016/j.jpsychires.2013.04.010. 23702251 10.1016/j.jpsychires.2013.04.010

[103] Huang R, Zhang Y, Bai Y, Han B, Ju M, Chen B, Yang L, Wang Y, Zhang H, Zhang H, Xie C, Zhang Z, Yao H. N6-Methyladenosine Modification of Fatty Acid Amide Hydrolase Messenger RNA in Circular RNA STAG1-Regulated Astrocyte Dysfunction and Depressive-like Behaviors. Biol Psychiatry. 2020;88:392–404. 10.1016/j.biopsych.2020.02.018. 32387133 10.1016/j.biopsych.2020.02.018

[104] Ye N, Rao S, Du T, Hu H, Liu Z, Shen Y, Xu Q. Intergenic variants may predispose to major depression disorder through regulation of long non-coding RNA expression. Gene. 2017;601:21–6. 10.1016/j.gene.2016.11.041. 27940106 10.1016/j.gene.2016.11.041

[105] Ni X, Liao Y, Li L, Zhang X, Wu Z. Therapeutic role of long non-coding RNA TCONS_00019174 in depressive disorders is dependent on Wnt/β-catenin signaling pathway. J Integr Neurosci. 2018;17:125–32. 10.31083/JIN-170052. 29036835 10.31083/JIN-170052

[106] Wang Z, Meng Z, Chen C. Screening of potential biomarkers in peripheral blood of patients with depression based on weighted gene co-expression network analysis and machine learning algorithms. Front Psychiatry. 2022;13:1009911. 10.3389/fpsyt.2022.1009911. 36325528 10.3389/fpsyt.2022.1009911 PMC9621316

[107] Horvath S. DNA methylation age of human tissues and cell types. Genome Biol. 2013;14:R115. 10.1186/gb-2013-14-10-r115. 24138928 10.1186/gb-2013-14-10-r115 PMC4015143

[108] Levine ME, Lu AT, Quach A, Chen BH, Assimes TL, Bandinelli S, Hou L, Baccarelli AA, Stewart JD, Li Y, Whitsel EA, Wilson JG, Reiner AP, Aviv A, Lohman K, Liu Y, Ferrucci L, Horvath S. An epigenetic biomarker of aging for lifespan and healthspan. Aging. 2018;10:573–91. 10.18632/aging.101414. 29676998 10.18632/aging.101414 PMC5940111

[109] Lu AT, Quach A, Wilson JG, Reiner AP, Aviv A, Raj K, Hou L, Baccarelli AA, Li Y, Stewart JD, Whitsel EA, Assimes TL, Ferrucci L, Horvath S. DNA methylation GrimAge strongly predicts lifespan and healthspan. Aging. 2019;11:303–27. 10.18632/aging.101684. 30669119 10.18632/aging.101684 PMC6366976

[110] Protsenko E, Yang R, Nier B, Reus V, Hammamieh R, Rampersaud R, Wu GWY, Hough CM, Epel E, Prather AA, Jett M, Gautam A, Mellon SH, Wolkowitz OM. GrimAge, an epigenetic predictor of mortality, is accelerated in major depressive disorder. Transl Psychiatry. 2021;11:193. 10.1038/s41398-021-01302-0. 33820909 10.1038/s41398-021-01302-0 PMC8021561

[111] Shindo R, Tanifuji T, Okazaki S, Otsuka I, Shirai T, Mouri K, Horai T, Hishimoto A. Accelerated epigenetic aging and decreased natural killer cells based on DNA methylation in patients with untreated major depressive disorder. NPJ Aging. 2023;9:19. 10.1038/s41514-023-00117-1. 37673891 10.1038/s41514-023-00117-1 PMC10482893

[112] Xu EY, Green C, McCartney DL, Han LKM, Evans KL, Walker RM, Gadd DA, Steele D, Waiter G, Campbell A, Lawrie SM, Cole JH, McIntosh AM, Shen X, Whalley HC. Epigenetic and Structural Brain Aging and Their Associations With Major Depressive Disorder. Biol Psychiatry Glob Open Sci. 2025;5:100577. 10.1016/j.bpsgos.2025.100577. 40994835 10.1016/j.bpsgos.2025.100577 PMC12454886

[113] Liufu C, Pang T, Zheng H, Yang L, Yang L, Huang X, Qian Y, Chang S. Epigenetic Clocks Identify Harmful Epigenetic Aging Linked to Depression Severity and Cognitive Deficits in Major Depressive Disorder. Aging Dis. 2025. 10.14336/AD.2025.0781. 40901984 10.14336/AD.2025.0781 PMC13437127

[114] Zhang Y, Du L, Bai Y, Han B, He C, Gong L, Huang R, Shen L, Chao J, Liu P, Zhang H, Zhang H, Gu L, Li J, Hu G, Xie C, Zhang Z, Yao H. CircDYM ameliorates depressive-like behavior by targeting miR-9 to regulate microglial activation via HSP90 ubiquitination. Mol Psychiatry. 2020;25:1175–90. 10.1038/s41380-018-0285-0. 30413800 10.1038/s41380-018-0285-0 PMC7244405

[115] Mohammadi S, Beh-Pajooh A, Ahmadimanesh M, Amini M, Ghazi-Khansari M, Moallem SA, Hosseini R, Nourian YH, Ghahremani MH. Evaluation of DNA methylation in BDNF, SLC6A4, NR3C1 and FKBP5 before and after treatment with selective serotonin-reuptake inhibitor in major depressive disorder. Epigenomics. 2022;14:1269–80. 10.2217/epi-2022-0246. 36377555 10.2217/epi-2022-0246

[116] Olstad EW, Nordeng HME, Sandve GK, Lyle R, Gervin K. Effects of prenatal exposure to (es)citalopram and maternal depression during pregnancy on DNA methylation and child neurodevelopment. Transl Psychiatry. 2023;13:149. 10.1038/s41398-023-02441-2. 37147306 10.1038/s41398-023-02441-2 PMC10163054

[117] Yu X, Fan Z, Yang T, Li H, Shi Y, Ye L, Huang R. Plasma circRNA HIPK2 as a putative biomarker for the diagnosis and prediction of therapeutic effects in major depressive disorder. Clin Chim Acta. 2024;552:117694. 10.1016/j.cca.2023.117694. 38065380 10.1016/j.cca.2023.117694

[118] Sales AJ, Joca SRL. Antidepressant administration modulates stress-induced DNA methylation and DNA methyltransferase expression in rat prefrontal cortex and hippocampus. Behav Brain Res. 2018;343:8–15. 10.1016/j.bbr.2018.01.022. 29378290 10.1016/j.bbr.2018.01.022

[119] Fan L, Zeng P, Wang X, Mo X, Ma Q, Zhou X, Yuan N, Liu Y, Xue Z, Huang J, Li X, Ding J, Chen J. Xiaoyao Pills, a Chinese patent medicine, treats mild and moderate depression: A randomized clinical trial combined with DNA methylation analysis. Phytomedicine. 2024;130:155660. 10.1016/j.phymed.2024.155660. 38815407 10.1016/j.phymed.2024.155660

[120] Schmauss C. An HDAC-dependent epigenetic mechanism that enhances the efficacy of the antidepressant drug fluoxetine. Sci Rep. 2015;5:8171. 10.1038/srep08171. 25639887 10.1038/srep08171 PMC4313090

[121] Du J, Liu N, Ma L, Liu R, Zuo D, Lan X, Yang J, Wei W, Peng X, Yu J. Antidepressant effect of the novel histone deacetylase-5 inhibitor T2943 in a chronic restraint stress mouse model. Biomed Pharmacother. 2024;171:116176. 10.1016/j.biopha.2024.116176. 38242038 10.1016/j.biopha.2024.116176

[122] Ge K, Bai Z, Wang J, Li Z, Gao F, Liu S, Zhang L, Gao F, Xie C. Engineering EVs-Mediated mRNA Delivery Regulates Microglia Function and Alleviates Depressive-Like Behaviors. Adv Mater. 2025;37:e2418872. 10.1002/adma.202418872. 39838773 10.1002/adma.202418872

[123] Kronman H, Torres-Berrío A, Sidoli S, Issler O, Godino A, Ramakrishnan A, Mews P, Lardner CK, Parise EM, Walker DM, van der Zee YY, Browne CJ, Boyce BF, Neve R, Garcia BA, Shen L, Peña CJ, Nestler EJ. Long-term behavioral and cell-type-specific molecular effects of early life stress are mediated by H3K79me2 dynamics in medium spiny neurons. Nat Neurosci. 2021;24:667–76. 10.1038/s41593-021-00814-8. 33723435 10.1038/s41593-021-00814-8 PMC8216773

[124] Al-Kachak A, Di Salvo G, Fulton SL, Chan JC, Farrelly LA, Lepack AE, Bastle RM, Kong L, Cathomas F, Newman EL, Menard C, Ramakrishnan A, Safovich P, Lyu Y, Covington HE, Shen L, Gleason K, Tamminga CA, Russo SJ, Maze I. Histone serotonylation in dorsal raphe nucleus contributes to stress- and antidepressant-mediated gene expression and behavior. Nat Commun. 2024;15:5042. 10.1038/s41467-024-49336-4. 38871707 10.1038/s41467-024-49336-4 PMC11176395

[125] Funatsuki T, Ogata H, Tahara H, Shimamoto A, Takekita Y, Koshikawa Y, Nonen S, Higasa K, Kinoshita T, Kato M. Changes in Multiple microRNA Levels with Antidepressant Treatment Are Associated with Remission and Interact with Key Pathways: A Comprehensive microRNA Analysis. Int J Mol Sci. 2023;24:12199. 10.3390/ijms241512199. 37569574 10.3390/ijms241512199 PMC10418406

[126] Ogata H, Higasa K, Kageyama Y, Tahara H, Shimamoto A, Takekita Y, Koshikawa Y, Nonen S, Kato T, Kinoshita T, Kato M. Relationship between circulating mitochondrial DNA and microRNA in patients with major depression. J Affect Disord. 2023;339:538–46. 10.1016/j.jad.2023.07.073. 37467797 10.1016/j.jad.2023.07.073

[127] Piriyaprasath K, Hasegawa M, Iwamoto Y, Kamimura R, Yusuf ASH, Fujii N, Yamamura K, Okamoto K. Effects of treadmill running on anxiety- and craniofacial pain-like behaviors with histone H3 acetylation in the brain of mice subjected to social defeat stress. PLoS ONE. 2025;20:e0318292. 10.1371/journal.pone.0318292. 39869606 10.1371/journal.pone.0318292 PMC11771924

[128] Liu Z, Li L, Ma S, Ye J, Zhang H, Li Y, Sair AT, Pan J, Liu X, Li X, Yan S, Liu X. High-Dietary Fiber Intake Alleviates Antenatal Obesity-Induced Postpartum Depression: Roles of Gut Microbiota and Microbial Metabolite Short-chain Fatty Acid Involved. J Agric Food Chem. 2020;68:13697–710. 10.1021/acs.jafc.0c04290. 33151669 10.1021/acs.jafc.0c04290

[129] Carvalho Silva R, Martini P, Hohoff C, Mattevi S, Bortolomasi M, Menesello V, Gennarelli M, Baune BT, Minelli A. DNA methylation changes in association with trauma-focused psychotherapy efficacy in treatment-resistant depression patients: a prospective longitudinal study. Eur J Psychotraumatol. 2024;15:2314913. 10.1080/20008066.2024.2314913. 38362742 10.1080/20008066.2024.2314913 PMC10878335

[130] Zallar LJ, Dupont MB. Brain and Blood Biomarkers of Major Depressive Disorder: A Systematic Review. JAMA Psychiatry. 2026;83:414–25. 10.1001/jamapsychiatry.2025.4613. 41739484 10.1001/jamapsychiatry.2025.4613

[131] Edgar RD, Jones MJ, Meaney MJ, Turecki G, Kobor MS. BECon: a tool for interpreting DNA methylation findings from blood in the context of brain. Transl Psychiatry. 2017;7:e1187–1187. 10.1038/tp.2017.171. 28763057 10.1038/tp.2017.171 PMC5611738

[132] Wang J, Shi A, Lyu J. A comprehensive atlas of epigenetic regulators reveals tissue-specific epigenetic regulation patterns. Epigenetics. 2023;18:2139067. 10.1080/15592294.2022.2139067. 36305095 10.1080/15592294.2022.2139067 PMC9980636

[133] Baldrighi GN, Cavagnola R, Calzari L, Sacco D, Costantino L, Ferrara F, Gentilini D. Investigating the Epigenetic Landscape of Major Depressive Disorder: A Genome-Wide Meta-Analysis of DNA Methylation Data, Including New Insights into Stochastic Epigenetic Mutations and Epivariations. Biomedicines. 2024;12:2181. 10.3390/biomedicines12102181. 39457495 10.3390/biomedicines12102181 PMC11505239

[134] Dereix AE, Ledyard R, Redhunt AM, Bloomquist TR, Brennan KJ, Baccarelli AA, Hacker MR, Burris HH. Maternal anxiety and depression in pregnancy and DNA methylation of the NR3C1 glucocorticoid receptor gene. Epigenomics. 2021;13:1701–9. 10.2217/epi-2020-0022. 33215541 10.2217/epi-2020-0022 PMC8579934

[135] Sommerer Y, Ohlei O, Dobricic V, Oakley DH, Wesse T, Sedghpour Sabet S, Demuth I, Franke A, Hyman BT, Lill CM, Bertram L. A correlation map of genome-wide DNA methylation patterns between paired human brain and buccal samples. Clin Epigenetics. 2022;14:139. 10.1186/s13148-022-01357-w. 36320053 10.1186/s13148-022-01357-w PMC9628033

[136] Rocks D, Jaric I, Tesfa L, Greally JM, Suzuki M, Kundakovic M. Cell type-specific chromatin accessibility analysis in the mouse and human brain. Epigenetics. 2022;17:202–19. 10.1080/15592294.2021.1896983. 33775205 10.1080/15592294.2021.1896983 PMC8865312

[137] Chan RF, Turecki G, Shabalin AA, Guintivano J, Zhao M, Xie LY, van Grootheest G, Kaminsky ZA, Dean B, Penninx BWJH, Aberg KA. E.J.C.G. van den Oord, Cell Type-Specific Methylome-wide Association Studies Implicate Neurotrophin and Innate Immune Signaling in Major Depressive Disorder. Biol Psychiatry. 2020;87:431–42. 10.1016/j.biopsych.2019.10.014. 31889537 10.1016/j.biopsych.2019.10.014 PMC9933050

[138] Sun YV, Turner ST, Smith JA, Hammond PI, Lazarus A, Van De Rostyne JL, Cunningham JM, Kardia SLR. Comparison of the DNA methylation profiles of human peripheral blood cells and transformed B-lymphocytes. Hum Genet. 2010;127:651–8. 10.1007/s00439-010-0810-y. 20238126 10.1007/s00439-010-0810-y PMC2873107

[139] Poulter MO, Du L, Weaver ICG, Palkovits M, Faludi G, Merali Z, Szyf M, Anisman H. GABAA receptor promoter hypermethylation in suicide brain: implications for the involvement of epigenetic processes. Biol Psychiatry. 2008;64:645–52. 10.1016/j.biopsych.2008.05.028. 18639864 10.1016/j.biopsych.2008.05.028

[140] Xie Y, Chen L, Wang L, Liu T, Zheng Y, Si L, Ge H, Xu H, Xiao L, Wang G. Single-nucleus transcriptomic analysis reveals the relationship between gene expression in oligodendrocyte lineage and major depressive disorder. J Transl Med. 2024;22:109. 10.1186/s12967-023-04727-x. 38281050 10.1186/s12967-023-04727-x PMC10822185

[141] Perna L, Zhang Y, Matias-Garcia PR, Ladwig K-H, Wiechmann T, Wild B, Waldenberger M, Schöttker B, Mons U, Ihle A, Kliegel M, Schwettmann L, Peters A, Brenner H. Subjective mental health, incidence of depressive symptoms in later life, and the role of epigenetics: results from two longitudinal cohort studies. Transl Psychiatry. 2020;10:323. 10.1038/s41398-020-00997-x. 32958748 10.1038/s41398-020-00997-x PMC7506005

[142] Berretta E, Guida E, Forni D, Provenzi L. Glucocorticoid receptor gene (NR3C1) methylation during the first thousand days: Environmental exposures and developmental outcomes. Neurosci Biobehav Rev. 2021;125:493–502. 10.1016/j.neubiorev.2021.03.003. 33689802 10.1016/j.neubiorev.2021.03.003

[143] Zhou L, Ng HK, Drautz-Moses DI, Schuster SC, Beck S, Kim C, Chambers JC, Loh M. Systematic evaluation of library preparation methods and sequencing platforms for high-throughput whole genome bisulfite sequencing. Sci Rep. 2019;9:10383. 10.1038/s41598-019-46875-5. 31316107 10.1038/s41598-019-46875-5 PMC6637168

